# Predicting bone cancer drugs properties through topological indices and machine learning

**DOI:** 10.1038/s41598-025-16497-1

**Published:** 2025-08-24

**Authors:** W. Eltayeb Ahmed, Muhammad Farhan Hanif, Ebraheem Alzahrani, Osman Abubakar Fiidow

**Affiliations:** 1 Department of Mathematics and Statistics, College of Science, Imam Mohammad Ibn Saud Islamic University (IMSIU), Riyadh, Saudi Arabia; 2 Department of Mathematics and Statistics, The University of Lahore, Lahore Campus, Lahore, Pakistan; 3 Department of Mathematics, Faculty of Science, King Abdulaziz University, Jeddah, Saudi Arabia; 4 Department of Public Health, Faculty of Health Science, Salaam University, Mogadishu, Somalia

**Keywords:** Chemical graph theory, QSPR, Neighborhood degree, Topological indices, Regression models, Bone cancer drugs, Random forest, Chemistry, Mathematics and computing

## Abstract

Chemical graph theory and topological indices are key tools in the study of molecular structures and their properties. This research explores anticancer drugs using neighborhood degree-based topological indices and compares their efficacy through regression and machine learning models. The QSPR approach is applied to 15 anticancer drugs by constructing neighborhood-based molecular graphs, and calculating their respective topological indices. Regression models like quadratic, cubic, and random forest are employed to predict response metrics including like boiling point, refractivity, and surface area of the drugs. Comparative studies indicate that quadratic models provide better predictive performance then their cubic counterparts in most scenarios. Random forest models also demonstrate satisfactory accuracy with smaller error bounds. The present findings highlight the usefulness of topological indices in chemoinformatics and their application in predicting drug response.

## Introduction

Graph theory is a discrete branch of mathematics that deals with graph models representing pairwise relationship between objects. A graph $G = (V, E)$ is consists of a set of vertices $V$ and a set of edges $E$, where an edge is a connection between a pair of vertices. The degree of a vertex $v \in V$, denoted by $\delta _v$, is the number of edges incident to $v$. In the case of simple graphs (i.e., no loops or multiple edges), corresponds to the number of vertices adjacent to $v$. The neighborhood of a vertex $v$ is the set of vertices that are connected to $v$, that is, $N(v) = \{ u \in V \mid \{u,v\} \in E \}$. The neighborhood degree sum of $v$ is defined as the sum of the degrees of all vertices in N(v), expressed as: $S(v) = \sum _{u \in N(v)}\delta _u$. This measure gprovides insight into the connectivity and influence of a vertex within its local environment^1^.

Now, consider a graph $G = (V, E)$ to be a simple connected graph, where $V(G)$ denotes the set of vertices and $E(G)$ the set of edges. For any vertex $u$ in $V(G)$, we define $N(u)$ as the open neighborhood of $u$, i.e., the set of vertices adjacent to $u$:

$$N(u) = \{ v \in V(G) \mid \{u,v\} \in E(G) \}.$$

The degree of a vertex in a neighborhood, denoted by $\delta (u)$, is defined as

$$\delta (u) = |N(N(u))| = \left| \bigcup _{v \in N(u)} N(v) \right|$$

This counts how many different vertices are neighbors to the neighbors of $u$, without including $u$ itself if it happens to be a neighbor to a neighbor. For any edge $uv \in E(G)$, define $\delta _u = \delta (u)$ and $\delta _v = \delta (v)$. These values are used to define various neighborhood-based topological indices. The degree of a vertex is the number of edges that it is connected to it. In a directed graph, it is important to distinguish between the in-degree (number of incoming edges) and the out-degree (number of outgoing edges). The order of a graph refers to the number of vertices it contains, while the size of the graph denotes the number of edges. The neighborhood M-polynomial of a graph *G* is given by:

1 $$\begin{aligned} NM(G; x, y) = \sum _{i,j} m(i,j) x^i y^j, \end{aligned}$$

where *m*(*i*, *j*) represents the number of edges $uv \in E(G)$ such that the pair $\{\delta _u, \delta _v\} = \{i,j\}$.

The common form of a neighborhood degree-based topological index is given by:

2 $$\begin{aligned} I(G) = \sum _{uv \in E(G)} f(\delta _u, \delta _v), \end{aligned}$$

where $f(\delta _u, \delta _v)$ is a function employed to specify particular indices.

Neighborhood First Zagreb index:

3 $$\begin{aligned} M_1(G) = \sum _{uv \in E(G)} (\delta _u + \delta _v), \end{aligned}$$

this index computes the total degree sum across all the edges.

Neighborhood second Zagreb index:

4 $$\begin{aligned} M_2(G) = \sum _{uv \in E(G)} (\delta _u \delta _v), \end{aligned}$$

this is a product-based descriptor of neighborhood degrees.

Neighborhood forgotten index:

5 $$\begin{aligned} FN(G) = \sum _{uv \in E(G)} (\delta _u^2 + \delta _v^2), \end{aligned}$$

this index represents the sum of the squares of the neighborhood degrees.

Neighborhood Second Modified Zagreb index

6 $$\begin{aligned} M_2^{mm}(G) = \sum _{uv \in E(G)} \frac{1}{\delta _u \delta _v}, \end{aligned}$$

this is an inverse product-based index.

Third Negiborhood $ND_3$ index:

7 $$\begin{aligned} ND_3(G) = \sum _{uv \in E(G)} \delta _u \delta _v (\delta _u + \delta _v), \end{aligned}$$

this combines both the sum and product of neighborhood degrees.

Fifth Negiborhood $ND_5$ index:

8 $$\begin{aligned} ND_5(G) = \sum _{uv \in E(G)} \left[ \frac{\delta _u}{\delta _v} + \frac{\delta _v}{\delta _u} \right] , \end{aligned}$$

this index captures the ratio relations between neighborhood degrees.

Neighborhood Harmonic index:

9 $$\begin{aligned} NH(G) = \sum _{uv \in E(G)} \frac{2}{\delta _u + \delta _v}, \end{aligned}$$

it is derived from the harmonic mean of neighborhood degrees. Neighborhood inverse sum index

10 $$\begin{aligned} NI(G) = \sum _{uv \in E(G)} \frac{\delta _u \delta _v}{\delta _u + \delta _v}, \end{aligned}$$

this index reflects a balance between the product and the sum of neighborhood degrees. Various topological indices are presented in Table 1.

**Table 1:** Derivation of some neighborhood degree-based topological indices.

Topological index	*f*(*x*, *y*)	Derivation from *NM*(*G*)
$M_1$	$x + y$	$(D_x + D_y)(NM(G))|_{x=y=1}$
$M_2$	*xy*	$(D_x D_y)(NM(G))|_{x=y=1}$
*FN*	$x^2 + y^2$	$(D_x^2 + D_y^2)(NM(G))|_{x=y=1}$
$M_2^{mm}$	$\dfrac{1}{xy}$	$(S_x S_y)(NM(G))|_{x=y=1}$
$ND_3$	$xy(x+y)$	$D_x D_y(D_x + D_y)(NM(G))|_{x=y=1}$
$ND_5$	$x^2 + y^2 \over xy$	$(D_x S_y + S_x D_y)(NM(G))|_{x=y=1}$
*NH*	$\dfrac{2}{x+y}$	$2 S_x J(NM(G))|_{x=y=1}$
*NI*	$\dfrac{xy}{x+y}$	$S_x J D_x D_y(NM(G))|_{x=y=1}$

Where:

$$\begin{aligned} D_x(f(x,y))&= x \frac{\partial f(x,y)}{\partial x}, \quad D_y(f(x,y)) = y \frac{\partial f(x,y)}{\partial y}, \\ S_x(f(x,y))&= \int _0^x \frac{f(t,y)}{t} dt, \quad S_y(f(x,y)) = \int _0^y \frac{f(x,t)}{t} dt, \\ J(f(x,y))&= f(x,x). \end{aligned}$$

Chemical graph theory is a sub-specialty of mathematical chemistry that uses graph theory to model and analyze molecules. In chemical graph theory, atoms are vertices and chemical bonds are edges in molecular graphs, abstract representations of actual chemical compounds^2^. Through this abstraction, scientists are able to probe molecular properties, forecast chemical behavior, and design new materials through the use of mathematical tools. Chemical graph theory can uncover information regarding isomer enumeration, compound stability, and structure-based features like rings or branches. It is also used to study molecular similarity and classification. Through the translation of chemistry into graph-theoretical terms, chemical graph theory makes it possible to apply combinatorial methods and algorithmic techniques to address problems in cheminformatics, drug discovery, and nanotechnology.

Topological indices are structure-based numerical descriptors that are extensively applied in Quantitative Structure Activity Relationships (QSAR) and Quantitative Structure Property Relationships (QSPR). Topological indices reflect critical information regarding the molecular shape, size, branchability, and connectivity and are powerful tools in modeling biological activity, toxicity, or physical attributes. Topological indices are classified according to the structural features that are being measured, and include degree-based, distance-based, or eigenvalue-based^3^. Some of the oldest and best-established are the Wiener index and Zagreb indices. Chemists can use the correlation of the topological indices with experimental measurements to predict the attributes of untested chemicals. Topological indices therefore constitute a connection between theoretical graph models and applied chemical or pharmacological consequences in molecular investigations.

Tamilarasi and Balamurugan^4^ used QSPR and QSTR with topological descriptors to examine the pharmacokinetic and toxicological profiles of antifungal medications. Their research provides insight into structure activity relationships, improving predictive modeling for drug discovery. Ravi and Chidambaram^5^ studied QSPR prediction based on neighborhood degree-type topological descriptors for predicting the physicochemical and pharmacological activity of medications for Parkinson s disease. Their research demonstrated the predictive power of the descriptors in identifying structural effects on drug activity. Shi et al.^6^ investigated novel QSPR predictive methods based on topological indices to enhance the design of cancer therapeutics. They demonstrated the usefulness of topological descriptors in correlating molecular structure with anticancer activity. Kara et al.^7^ studied COVID-19 medications using QSPR models based on neighborhood eccentricity-topological indices. Their comparative study tested the effectiveness of these indices in predicting the therapeutic properties of SARS-CoV-2 compounds.

Awan et al.^8^ utilized a paradigmatic approach with topological descriptors in modeling babesiosis medications through QSPR analysis. Their approach focused on estimating significant physical properties of these antiprotozoal drugs. Sardar et al.^9^ developed enhanced QSAR methodologies for predicting key drug properties by integrating topological indices with advanced machine learning models. Their method aimed to improve accuracy and rigor in structure activity relationship predictions in drug discovery. HH et al.^10^ applied topological indices in QSPR/QSAR analysis for Alzheimer s disease therapeutic candidates, aiming to correlate molecular descriptors with relevant properties for evaluating potential treatments. Arockiaraj et al.^11^ comparatively studied degree-, neighborhood-, and reverse degree-based topological indices for building QSPR models of anticancer agents used in treating lung cancer. They tested the predictive ability of these indices in forecasting key physicochemical and biological descriptors of anticancer molecules.

Das et al.^12^ calculated topological indices for Molnupiravir and its analog antiviral agents for COVID-19 treatment using QSPR modeling, underlining the importance of graph theory in drug evaluation. Neighborhood degree sum-based indices for silicon carbide networks, proposed by Das and Kumar^13^, allowed for comparative structural analysis to improve understanding of molecular property prediction.

Huang et al.^14^ conducted QSPR prediction of glaucoma drugs based on topological descriptors coupled with XGBoost and regression methods. Their contribution presents a successful property prediction with machine learning in biomedical fields. Qin et al.^15^ studied QSPR of drugs against pulmonary cancer based on topological indices via a Python QSPR system. This study validates the predictive power of graph-theoretical features in oncology drug assessment. Qin et al.^16^ utilized topological indices in a Python framework to predict some properties of anti-arrhythmia drugs. Their method is illustrated to have reliability and interpretability in molecular-structure based drug prediction. AlQadi et al.^17^ had treated by statistical means the molecular network of phthalocyanine applying such a topological characteristic. The correlation between the structural motifs and the physicochemical properties is particularly emphasized. Wei et al.^18^ used linear regression for testing QSPR models of some drugs, using topological descriptors. Their results corroborate the simplicity and performance of regression-based techniques for drug property prediction. Yang et al.^19^ studied (2D) covalent organic frameworks with these and derived topological properties. Their theoretical analysis justifies the polynomial representation of molecular graphs.

## Bone drugs

Actinomycin D is used as an antineoplastic antibiotic that intercalates into DNA, thereby preventing RNA synthesis. It is primarily employed to treat gestational trophoblastic neoplasia, Wilms tumor, and rhabdomyosarcoma. It interferes with transcription by binding to the DNA double helix and blocking RNA polymerase mobility. It is administered intravenously, usually as part of combination chemotherapy. Common adverse effects include bone marrow suppression, vomiting, alopecia, and nausea^20^. Due to its high toxicity, dosing must be precise and closely monitored. Resistance may occur through increased efflux or alterations in drug targets. While its cell cycle-arresting capabilities contribute to its effectiveness, it must be used with caution, as it can cause tissue necrosis if extravasation occurs during administration.

Anastrozole is a nonsteroidal aromatase inhibitor primarily used in postmenopausal women to treat hormone receptor-positive breast cancer. It suppresses the action of the enzyme aromatase, which converts androgens to estrogens, thereby lowering estrogen levels that promote certain breast cancers. Anastrozole is administered orally, typically once daily. Common side effects include hot flashes, joint pain, weakness, and decreased bone density. Unlike tamoxifen, it does not increase the risk of uterine cancer or thrombosis^21^. It is commonly used as adjuvant therapy following surgery and can also be used in metastatic disease. Anastrozole is well tolerated and highly effective in significantly reducing the risk of recurrence. It is contraindicated in premenopausal women due to its estrogen-reducing mechanism.

Cabozantinib is a multi-kinase inhibitor that targets MET, VEGFR, RET, and other tumor-associated tyrosine kinases involved in tumor growth and angiogenesis. It is prescribed for advanced renal cell carcinoma, hepatocellular carcinoma, and medullary thyroid carcinoma. Administered orally, it inhibits pathways involved in cancer cell proliferation and angiogenesis. Adverse effects include diarrhea, fatigue, hypertension, hand-foot syndrome, and elevated liver enzyme levels^22^. It has shown efficacy in delaying disease progression and improving survival in various cancers. Dosage adjustments may be necessary due to toxicity. Since it interacts with CYP3A4 inhibitors and inducers, monitoring is required. Cabozantinib is a powerful targeted therapy that disrupts cancer cell survival and metastatic signaling pathways.

Cyclophosphamide is used in numerous cancers such as ovarian cancer, breast cancer, leukemias, and lymphomas as an alkylating agent. It cross-links DNA and results in cell cycle arrest and apoptosis in rapidly dividing cell populations. It is administered intravenously or orally as a prodrug activated in the liver^23^. The major toxicities are alopecia, bone marrow depression, hemorrhagic cystitis, nausea, and vomiting. Mesna is usually co-administered as a protector of the urinary bladder. It also finds application in the immunosuppressive treatment of autoimmune conditions such as lupus and multiple sclerosis. The oncological versatility of the drug arises from its wide range of activity against various cancers. Long-term treatment is risky and increases the likelihood of secondary cancers as well as infertility. Monitoring blood counts and hydration status is important to reduce toxicity and enhance drug efficacy.

Doxorubicin is an anthracycline antibiotic used in the treatment of several cancers, including breast cancer, lymphomas, and sarcomas. It intercalates DNA and causes DNA strand breaks through inhibition of topoisomerase II^24^. It also generates free radicals, contributing to its cellular toxicity. Doxorubicin is administered intravenously and possesses strong antitumor activity. The primary concern is cardiotoxicity, especially at cumulative doses, which is usually monitored via echocardiograms. Myelosuppression, vomiting, alopecia, and mucositis are other common adverse effects. Extravasation can cause tissue necrosis. Liposomal formulations are used to reduce cardiac risk. Doxorubicin remains a mainstay in chemotherapy regimens, although careful dosing and monitoring are essential to prevent severe adverse effects, such as irreversible myocardial damage.

Etoposide is a topoisomerase II inhibitor used in the treatment of small-cell lung carcinoma, testicular cancer, lymphomas, and leukemia. It inhibits DNA re-ligation during replication, leading to DNA strand breaks and cell death. Both intravenous and oral forms are available, and it is typically used in combination chemotherapy regimens. Myelosuppression, nausea, alopecia, and hypotension are frequent toxicities^25^. It is cell cycle-specific, acting mainly in the G2 and S phases. The efficacy of etoposide depends on dose and schedule, with drug efflux or enzyme changes occasionally leading to resistance. Close monitoring of blood counts and renal function is essential. Its inclusion in combination protocols makes it valuable in treating aggressive and resistant neoplasms.

Gemcitabine is a nucleoside analog used to treat pancreatic, lung, bladder, and breast cancers. Once incorporated into DNA, it blocks further DNA synthesis and induces apoptosis. It is administered intravenously as a prodrug, which is converted intracellularly into active metabolites^26^. Adverse effects include myelosuppression, influenza-like syndrome, rash, and mild gastrointestinal disturbances. It is usually combined with other chemotherapeutic agents for synergistic effects. The relatively low toxicity of gemcitabine allows for treatment of frail or elderly patients. It is widely used in treatment protocols due to its efficacy against solid tumors. Dosing regimens are adjusted based on the cancer type, and periodic monitoring of blood counts and liver function is necessary to ensure safety and efficacy.

Ibandronate is one of the bisphosphonates mainly used to treat and prevent osteoporosis and breast cancer metastases to bone. It inhibits bone resorption mediated by osteoclasts, resulting in increased bone mineral density. It is administered either orally or intravenously to decrease fractures and skeletal-related event risk. Gastrointestinal discomfort, flu-like syndrome, and osteonecrosis of the jaw are some of the adverse effects, though very rare^27^. Ibandronate can also be used to treat hypercalcemia of malignancy. It has a long bone tissue half-life, so dosing can be done once monthly. Proper administration (with water, while sitting or standing) is important to avoid esophageal irritation. The anti-resorptive action of ibandronate is useful in the management of both oncologic and metabolic bone diseases.

Ifosfamide is an alkylating agent most notably used in the treatment of testicular cancer, sarcomas, and lymphomas. It acts by inhibiting DNA replication, leading to the death of rapidly proliferating cancer cells. Administered intravenously, Ifosfamide is sometimes co-administered with other agents in chemotherapy to ensure greater efficacy^28^. Co-administration with mesna is necessary to prevent hemorrhagic cystitis, a toxicity affecting the bladder. Typical side effects include nausea, vomiting, bone marrow depression, and neurotoxicity. Hydration is also necessary during administration. Monitoring renal and hepatic function is required. Treatment with Ifosfamide is reserved for specifically selected patients based on the toxicity it can induce, especially central nervous system toxicity.

Letrozole is a nonsteroidal aromatase inhibitor most commonly used in postmenopausal women with hormone receptor-positive breast cancer. It acts by inhibiting the enzyme aromatase, which converts androgens into estrogens, thereby decreasing estrogen levels and inhibiting cancer progression^29^. It is administered orally, typically once a day. It is also used off-label to induce fertility in women with polycystic ovary syndrome (PCOS). Side effects can include hot flashes, tiredness, arthralgia, and an increased risk of osteoporosis due to reduced estrogen levels. Letrozole is highly valuable in both adjuvant and metastatic settings, particularly in hormone-sensitive breast cancer, and is recommended in current clinical guidelines. Long-term use may require bone density monitoring.

Pazopanib is an oral multi-target tyrosine kinase inhibitor (TKI) primarily used to manage advanced renal cancer and soft tissue sarcoma. It inhibits VEGFR, PDGFR, and c-KIT, thereby preventing angiogenesis and tumor growth. Pazopanib, taken once daily, is clinically beneficial in delaying disease progression^30^. Side effects include hypertension, diarrhea, fatigue, elevated liver enzymes, and hair color changes. Liver function tests should be monitored regularly due to the risk of hepatotoxicity. Pazopanib provides an alternative among available TKIs with an acceptable toxicity profile. However, it may interact with drugs metabolized by CYP3A4 and should be used cautiously in patients with hepatic or cardiovascular disease.

Regorafenib is an oral multi-target kinase inhibitor approved for metastatic colorectal cancer, gastrointestinal stromal tumors (GIST), and hepatocellular carcinoma. It targets VEGFR, PDGFR, FGFR, RAF, and several other kinases involved in tumor angiogenesis, oncogenesis, and the tumor microenvironment. Regorafenib is taken daily for three weeks in a four-week cycle and helps delay disease progression in patients who have failed prior therapies^31^. Side effects include hand-foot skin reaction, fatigue, diarrhea, hypertension, and hepatotoxicity. Blood pressure and liver function must be regularly monitored. Regorafenib s broad kinase inhibition results in numerous drug interactions. It is an essential option for patients with limited therapeutic alternatives and requires careful toxicity management.

Sorafenib is an oral multi-kinase inhibitor mainly used to treat advanced differentiated thyroid carcinoma, renal cell carcinoma, and hepatocellular carcinoma. It inhibits tumor progression and angiogenesis by targeting RAF kinase, VEGFR, PDGFR, and other kinases. It is administered twice daily and is metabolized by the liver via CYP3A4. Common adverse effects include rash, diarrhea, hypertension, and hand-foot skin reaction^32,33^. Liver function tests are essential during treatment, especially in patients with hepatic impairment. Sorafenib improves survival in several cancers and is a standard therapy for hepatocellular carcinoma. Dosage adjustments may be needed due to side effects, and patient tolerance varies significantly.

Tamoxifen is a selective estrogen receptor modulator (SERM) extensively employed in the treatment and prevention of breast cancer that is estrogen receptor-positive. Tamoxifen competes with estrogen for binding to the estrogen receptors in breast tissue, thereby blocking the proliferative effects of estrogen. It is administered orally, typically over a course of 5 to 10 years as adjuvant therapy^34^. It decreases the risk of breast cancer in individuals at high risk. Common side effects include hot flushes, vaginal dryness, mood swings, and an elevated risk of thromboembolic events and endometrial cancer. Despite these risks, tamoxifen has significantly improved survival rates and reduced recurrence. Regular follow-up for endometrial pathology and blood clot monitoring is advised during long-term therapy.

Zoledronic acid is a potent intravenous bisphosphonate used to treat metastatic bone disease, hypercalcemia of malignancy, and osteoporosis. It inhibits osteoclast activity, thereby increasing bone strength and reducing skeletal-related events. It is administered every 3 4 weeks in cancer patients or yearly for osteoporosis. Zoledronic acid significantly reduces bone pain and fracture rates in patients with metastatic cancer. Common adverse effects include flu-like symptoms, hypocalcemia, and renal dysfunction^35^. A serious but rare adverse effect is osteonecrosis of the jaw, especially in patients undergoing dental procedures. Renal function should be evaluated before each administration, and calcium/vitamin D supplementation should be adequate.

**Fig. 1:**
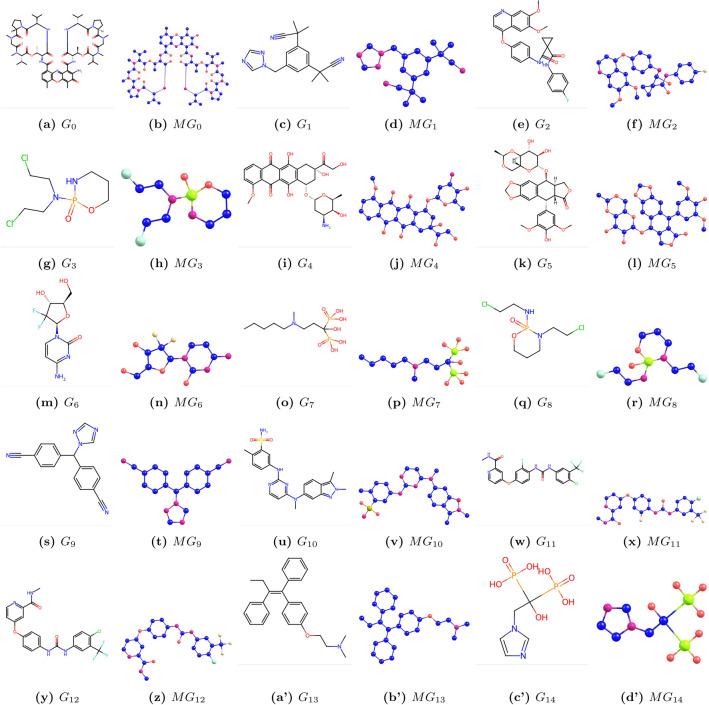
Graphs $G_i$ and corresponding molecular graphs $MG_i$ of bone drugs ($i=0,1,\dots ,14$).

We denote chemical structure as $G_i$, where $i=0,1,...14$, and molecular structure as $MG_i$, where $i=0,1,...14$. Chemical and molecular structures are shown in Fig. 1. The physicochemical properties are listed in Table 2.

**Table 2:** Physiochemical properties.

Graphs	Drug	BP	EV	FP	MR	SA	P	ST	MV
$G_{0}$	*ActinomycinD*	1386	211.5	792.1	323.7	356	128.3	53.9	880.7
$G_{1}$	*Anastrozole*	469.7	73.2	237.9	90	78	35.7	42.2	270.3
$G_{2}$	*Cabozantinib*	758.1	110.4	412.3	137	99	54.3	67.5	359
$G_{3}$	*Cyclophosphamide*	336.1	57.9	157.1	58.1	51	23	44.3	195.3
$G_{4}$	*Doxorubicin*	810.3	123.5	443.8	131.5	206	52.1	96.4	336.6
$G_{5}$	*Etoposide*	798.1	121.7	263.6	140.1	161	55.5	76.5	378.5
$G_{6}$	*Gemcitabine*	482.7	86.2	245.7	52.1	108	20.6	65.3	142.3
$G_{7}$	*Ibandronate*	587.8	100.7	309.3	68.9	158	27.3	70.8	220.2
$G_{8}$	*Ifosfamide*	336.1	57.9	157.1	58.1	51	23	44.3	195.7
$G_{9}$	*Letrozole*	563.5	84.7	294.6	87.1	78	34.5	53.5	234.5
$G_{10}$	*Pazopanib*	728.8	106.4	394.6	120.2	127	47.6	56.7	310.4
$G_{11}$	*Regorafenib*	513.4	78.5	264.3	113.1	92	44.8	50.8	323.7
$G_{12}$	*Sorafenib*	523.3	79.7	270.3	113.1	92	44.8	51.8	319.5
$G_{13}$	*Tamoxifen*	482.3	74.7	140	118.9	12	47.1	40.4	356.2
$G_{14}$	*Zoledronicacid*	764	116.7	415.8	50.3	173	19.9	149.7	127.4

### Theorem 1

Let $G_0$ be the molecular graph of *Actinomycin D*, then the neighborhood M-polynomial is

$$NM(G_{0};x,y)=x^{2} y^{4} \left( 2 x^{5} y^{4} + 4 x^{4} y^{4} + 4 x^{4} y^{2} + 2 x^{3} y^{3} + 2 x^{3} y^{2} + 4 x^{2} y^{3} + 2 x^{2} y + x^{2} + 2 y\right) .$$

### Proof

We consider the molecular graph of *Actinomycin D* and apply the neighborhood degree-based sum partition of its edges in the following form: $|E_1{(2,5)}|$ =2, $|E_2{(5,7)}|$ =2, $|E_3{(4,7)}|$ =4, $|E_4{(7,8)}|$ =2, $|E_5{(6,8)}|$ =4, $|E_6{(6,6)}|$ =4, $|E_7{(5,6)}|$ =2, $|E_8{(4,5)}|$ =2, $|E_9{(4,4)}|$ =1,

Using the definition in equation (1), we substitute the partition of the edges into the equation and simplify. This gives:

$$\begin{aligned} NM(G_{0};x,y)= & \sum _{2\le 5}\lambda _{(2,5)}x^{2}y^{5} +\sum _{5\le 7}\lambda _{(5,7)}x^{5}y^{7} +\sum _{4\le 7}\lambda _{(4,7)}x^{4}y^{7} +\sum _{7\le 8}\lambda _{(7,8)}x^{7}y^{8} +\sum _{6\le 8}\lambda _{(6,8)}x^{6}y^{8}\\+ & \sum _{6\le 6}\lambda _{(6,6)}x^{6}y^{6} +\sum _{5\le 6}\lambda _{(5,6)}x^{5}y^{6} +\sum _{4\le 5}\lambda _{(4,5)}x^{4}y^{5} +\sum _{4\le 4}\lambda _{(4,4)}x^{4}y^{4}\\ M(G_{0};x,y)= & x^{2} y^{4} \left( 2 x^{5} y^{4} + 4 x^{4} y^{4} + 4 x^{4} y^{2} + 2 x^{3} y^{3} + 2 x^{3} y^{2} + 4 x^{2} y^{3} + 2 x^{2} y + x^{2} + 2 y\right) . \end{aligned}$$

$$\square$$

### Theorem 2

Some topological indices for $G_{0}=Actinomycin D$ are

$M_1$ =264, $M_2$ =17100, *FN* =1616, $M_2^{nm}$ =18.9039, $ND_3$ =9456, $ND_5$ =49.85, *NH* =4.1949, *NI* =63.9522.

### Proof

The NM-polynomial obtained in Theorem 1 and Table 1 is used to compute the indices, as:

$$\begin{aligned} Dx(NM)NM(G_{0};x,y)&= x^{2}y^{4} \left( 14 x^{5} y^{4} + 24 x^{4} y^{4} + 24 x^{4} y^{2} + 10 x^{3} y^{3} + 10 x^{3} y^{2} + 16 x^{2} y^{3} + 8 x^{2} y + 4 x^{2} + 4 y\right) ,\\ Dy(NM)NM(G_{0};x,y)&= x^{2} y^{4} \left( 16 x^{5} y^{4} + 32 x^{4} y^{4} + 24 x^{4} y^{2} + 14 x^{3} y^{3} + 12 x^{3} y^{2} + 28 x^{2} y^{3} + 10 x^{2} y + 4 x^{2} + 10 y\right) ,\\ Dx^2(NM)NM(G_{0};x,y)&= x^{2} y^{4} \left( 98 x^{5} y^{4} + 144 x^{4} y^{4} + 144 x^{4} y^{2} + 50 x^{3} y^{3} + 50 x^{3} y^{2} + 64 x^{2} y^{3} + 32 x^{2} y + 16 x^{2} + 8 y\right) ,\\ Dy^2(NM)NM(G_{0};x,y)&= x^{2} y^{4} \left( 128 x^{5} y^{4} + 256 x^{4} y^{4} + 144 x^{4} y^{2} + 98 x^{3} y^{3} + 72 x^{3} y^{2} + 196 x^{2} y^{3} + 50 x^{2} y + 16 x^{2} + 50 y\right) ,\\ Sx(NM)NM(G_{0};x,y)&= \frac{1}{420}[x^{2} y^{4} \left( 120 x^{5} y^{4} + 280 x^{4} y^{2} \left( y^{2} + 1\right) + 168 x^{3} y^{2} \left( y + 1\right) + 105 x^{2} \left( 4 y^{3} + 2 y + 1\right) + 420 y\right) ],\\ Sy(NM)NM(G_{0};x,y)&= \frac{1}{420}[x^{2} y^{4} \left( 105 x^{4} y^{4} \left( x + 2\right) + 140 x^{3} y^{2} \left( 2 x + 1\right) + 120 x^{2} y^{3} \left( x + 2\right) + 105 x^{2} + 168 y \left( x^{2} + 1\right) \right) ],\\ J(NM)NM(G_{0};x,y)&= x^{7} \left( 2 x^{8} + 4 x^{7} + 6 x^{5} + 6 x^{4} + 2 x^{2} + x + 2\right) . \end{aligned}$$

$$\square$$

The NM-polynomials for other drug structures from $G_{1}$ to $G_{14}$ can be obtained similarly to the proof of Theorem 1 and are shown in Table 3.

$G_{1}$= Anastrozole; $G_{2}$= Cabozantinib; $G_{3}$= Cyclophosphamide; $G_{4}$= Doxorubicin; $G_{5}$= Etoposide; $G_{6}$= Gemcitabine; $G_{7}$= Ibandronate; $G_{8}$= Ifosfamide; $G_{9}$= Letrozole; $G_{10}$= Pazopanib; $G_{11}$= Regorafenib; $G_{12}$= Sorafenib; $G_{13}$= Tamoxifen; $G_{14}$= Zoledronic acid; we have:

$$\begin{aligned} NM(G_1;x,y)&= x^{2} y^{4}(2 x^{5} y^{4} + 4 x^{4} y^{4} + 4 x^{4} y^{2} + 2 x^{3} y^{3} + 2 x^{3} y^{2} + 4 x^{2} y^{3} + 2 x^{2} y + x^{2} + 2 y),\\ NM(G_2;x,y)&= x^{2} y^{4}(2 x^{5} y^{6} + 2 x^{5} y^{4} + x^{5} y^{3} + 2 x^{4} y^{6} + x^{4} y^{4} + 6 x^{4} y^{3} + 4 x^{4} y^{2} + 2 x^{3} y^{3} + 6 x^{3} y^{2} + 6 x^{3} y \\&+ 2 x^{2} y^{3} + 2 x^{2} y + 2 x y^{3} + x y + 2),\\ NM(G_3;x,y)&= x^{2} y^{3} (x^{6} y^{5} + 2 x^{4} y^{5} + 2 x^{3} y^{5} + x^{2} y^{5} + 2 x^{2} y^{3} + 2 x^{2} y + 2 x y^{2} + 2),\\ NM(G_4;x,y)&= x^{2} y^{4} (x^{7} y^{5} + 2 x^{6} y^{5} + 8 x^{5} y^{5} + 6 x^{5} y^{4} + x^{5} y^{3} + 3 x^{4} y^{3} + 5 x^{4} y^{2} + x^{3} y^{4} + x^{3} y^{3} + x^{2} y^{4} + 2 x^{2} y^{3} + 2 x^{2} y\\&+ 6 x y^{3} + 2 x y^{2} + 2),\\ NM(G_5;x,y)&= x^{2} y^{4} (x^{7} y^{5} + 2 x^{6} y^{5} + 3 x^{6} y^{4} + x^{5} y^{5} + 2 x^{5} y^{4} + 5 x^{5} y^{3} + x^{4} y^{5} + 4 x^{4} y^{4} + 9 x^{4} y^{3} + x^{3} y^{4} + 3 x^{3} y^{3}\\&+ 2 x^{3} y^{2} + 3 x^{3} y + 2 x^{2} y^{3} + 2 x^{2} y + 3 x y^{3} + x y^{2} + x y + 2),\\ NM(G_6;x,y)&= x^{2} y^{4} (2 x^{6} y^{5} + x^{6} y^{4} + x^{5} y^{4} + x^{4} y^{5} + x^{4} y^{4} + x^{4} y^{3} + x^{4} y^{2} + x^{3} y^{4} + x^{3} y^{2} + 2 x^{3} y + 2 x^{2} y^{4} + x^{2} y^{3}\\&+ x y^{4} + x y^{2} + x y + 1),\\ NM(G_7;x,y)&= x^{2} y^{3} (2 x^{5} y^{8} + x^{4} y^{8} + x^{3} y^{3} + 2 x^{3} y^{2} + x^{2} y^{8} + 6 x^{2} y^{4} + x^{2} y^{2} + x^{2} y + x y^{2} + x y + 1),\\ NM(G_8;x,y)&= x^{2} y^{3} (x^{6} y^{5} + 2 x^{4} y^{5} + 2 x^{3} y^{5} + x^{2} y^{5} + 2 x^{2} y^{3} + x^{2} y^{2} + x^{2} y + x y^{2} + x y + 2),\\ NM(G_9;x,y)&= x^{2} y^{4} (3 x^{5} y^{5} + 6 x^{3} y^{3} + 4 x^{3} y^{2} + 4 x^{3} y + 2 x^{2} y^{2} + 2 x^{2} y + x^{2} + 2),\\ NM(G_{10};x,y)&= x^{3} y^{5} (2 x^{4} y^{3} + 2 x^{4} y^{2} + 3 x^{3} y^{4} + 5 x^{3} y^{2} + 5 x^{3} y + x^{2} y^{3} + 2 x^{2} y^{2} + 3 x^{2} y + 2 x^{2} + 3 x y + 2 x + 2 y^{2} + 2 y),\\ NM(G_{11};x,y)&= x^{2} y^{4}(3 x^{4} y^{5} + 4 x^{4} y^{3} + 7 x^{4} y^{2} + 2 x^{3} y^{3} + 6 x^{3} y^{2} + 2 x^{3} y + 4 x^{2} y^{2} + 2 x^{2} y + 3 x y^{2} + x y + 1),\\ NM(G_{12};x,y)&= x^{2} y^{4} (3 x^{4} y^{5} + 2 x^{4} y^{3} + 6 x^{4} y^{2} + x^{3} y^{3} + 9 x^{3} y^{2} + 3 x^{3} y + 4 x^{2} y^{2} + 2 x^{2} y + 2 x y^{2} + x y + 1),\\ NM(G_{13};x,y)&= x^{2} y^{4} (x^{6} y^{5} + 2 x^{5} y^{5} + x^{5} y^{4} + 6 x^{3} y^{3} + 3 x^{3} y^{2} + 2 x^{3} y + x^{2} y^{4} + 7 x^{2} y + 4 x^{2} + 2 x + 1),\\ NM(G_{14};x,y)&=x^{4} y^{4} (3 x^{3} y^{7} + x^{2} y^{3} + 2 x y^{2} + y^{7} + 6 y^{3} + 2 y + 1). \end{aligned}$$

**Table 3:** Different toplogical indices.

Graphs	Drug name	$M_1$	$M_2$	FN	$ND_3$	$ND_5$	NH	NI
$G_{0}$	*ActinomycinD*	1162	329461	7606	46982	212.1012	16.6743	278.8874
$G_{1}$	*Anastrozole*	264	17100	1616	9456	49.85	4.1949	63.9522
$G_{2}$	*Cabozantinib*	480	56816	3008	18274	87.0143	7.3764	116.9891
$G_{3}$	*Cyclophosphamide*	144	5040	848	4828	30.3167	3.0851	34.6277
$G_{4}$	*Doxorubicin*	550	74104	3816	25474	94.8679	7.1905	132.5252
$G_{5}$	*Etoposide*	604	90115	4052	26682	102.271	8.0812	147.4245
$G_{6}$	*Gemcitabine*	232	13132	1552	9792	42.2075	3.3285	55.5364
$G_{7}$	*Ibandronate*	202	9717	1330	7774	40.4366	3.5539	47.3827
$G_{8}$	*Ifosfamide*	144	5040	848	4844	30.1833	3.093	34.6892
$G_{9}$	*Letrozole*	262	16905	1536	8928	50.4429	4.6634	64.1327
$G_{10}$	*Pazopanib*	404	40228	2522	15046	72.3321	5.8765	98.357
$G_{11}$	*Regorafenib*	390	37496	2288	12932	74.0571	6.5007	95.1728
$G_{12}$	*Sorafenib*	374	34528	2160	12066	71.4952	6.3829	91.4764
$G_{13}$	*Tamoxifen*	316	24640	1810	10462	62.4611	6.0734	77.5096
$G_{14}$	*Zoledronicacid*	196	9163	1358	8360	35.8561	2.7695	46.1692

**Table 4:** Statistical parameters and regression models for $M_1(G)$.

Property	$\text {Model}$	$\text {Equation}$	*R*	$R^2$	$S_E$	*F*	$p\text {-value}$
BP	Quadratic	$y=343.2294+0.6512x+0.0002x^2$	0.910	0.829	0.448	29.069	0.000
	Cubic	$y=351.1209+0.5885x+0.0003x^2-0.00001x^3$	0.910	0.829	2.045	17.766	0.000
EV	Quadratic	$y=65.3044+0.0543x+0.0001x^2$	0.907	0.823	0.066	27.956	0.000
	Cubic	$y=73.6058-0.0117x+0.0002x^2-0.00001x^3$	0.908	0.824	0.302	17.180	0.000
FP	Quadratic	$y=211.1650+0.1014x+0.0003x^2$	0.847	0.718	0.358	15.292	0.001
	Cubic	$y=82.5299+1.1239x-0.0018x^2+0.00001x^3$	0.854	0.729	1.605	9.852	0.002
MR	Quadratic	$y=31.8702+0.1704x+0.0001x^2$	0.980	0.961	0.055	148.073	0.000
	Cubic	$y=-25.1515+0.6237x-0.0009x^2+0.00001x^3$	0.987	0.973	0.208	133.277	0.000
SA	Quadratic	$y=79.6755-0.0005x+0.0002x^2$	0.837	0.701	0.188	14.041	0.001
	Cubic	$y=154.2993-0.5937x+0.0015x^2-0.00001x^3$	0.845	0.714	0.840	9.167	0.003
*P*	Quadratic	$y=12.6089+0.0675x+0.00001x^2$	0.980	0.961	0.022	147.734	0.000
	Cubic	$y=-10.0119+0.2473x-0.0004x^2+0.00001x^3$	0.986	0.973	0.083	132.916	0.000
MV	Quadratic	$y=137.6573+0.2931x+0.0003x^2$	0.965	0.932	0.192	82.428	0.000
	Cubic	$y=-16.3735+1.5175x-0.0023x^2+0.00001x^3$	0.972	0.945	0.790	62.838	0.000

**Fig. 2:**
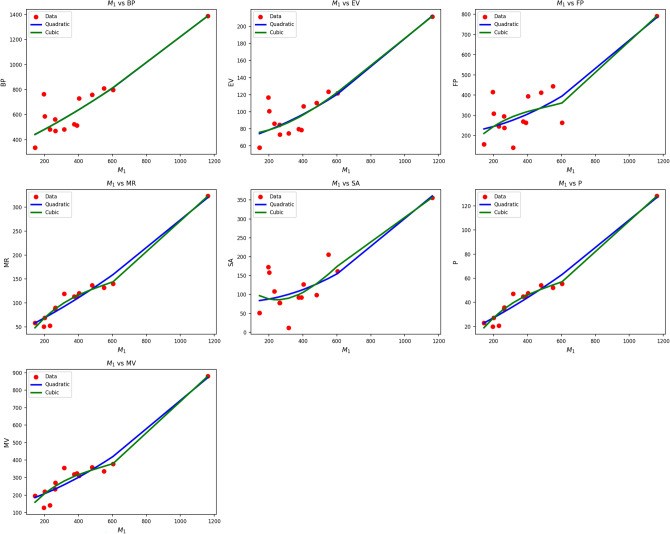
Scatter plots of actual data points (red) and regression model fits (Quadratic in blue, cubic in green) for various drug response parameters versus ${M_1(G)}$.

Table 4 also evaluates the association between M1(G) and drug-response metrics using quadratic and cubic regression models. Quadratic models systematically perform better than their cubic equivalents, as indicated by higher R values (0.837 0.974) and significantly greater $R^2$ values. Cubic BP, EV, and FP models are weak in comparison, with $R^2$ values as low as 0.184 for EV, implying limited explanatory capacity of this predictor in the cubic models. The standard error is also lower in almost all quadratic models, reflecting improved predictive accuracy.

F-statistics also add weight to the quadratic models’ reliability, with particularly high values observed in the cases of MR, P, and MV, implying strong general significance. All p-values of the quadratic models are well below 0.001, supporting their statistical validity, while some p-values of the cubic models reach or surpass 0.05, undermining their credibility. Figure 2 corroborates the statistical results through the visualization of regression fits over actual data points. The blue curves of the quadratics closely track the red data points, particularly in sequences such as MR, P, and MV, where the alignment is nearly perfect. The green curves of the cubics, conversely, tend to diverge unnecessarily, yielding poor fits in regions with low data density. This visual confirmation reinforces the numerical results, establishing that drug response as a function of $M_1(G)$ is better represented by quadratic models than by cubic ones due to improved fit and reduced error rates.

**Table 5:** Statistical parameters and regression models for $M_2(G)$.

Property	$\text {Model}$	$\text {Equation}$	*R*	$R^2$	$S_E$	*F*	$p\text {-value}$
BP	Quadratic	$y=437.4176+0.0050x-0.00001x^2$	0.909	0.826	0.002	28.513	0.000
	Cubic	$y=0.0000+0.0311x-0.0000x^2+0.00001x^3$	0.623	0.388	0.005	3.806	0.052
EV	Quadratic	$y=72.8146+0.0006x-0.00001x^2$	0.907	0.823	0.000	27.971	0.000
	Cubic	$y=0.0000+0.0048x-0.0000x^2+0.00001x^3$	0.429	0.184	0.001	1.352	0.295
FP	Quadratic	$y=232.5637+0.0017x-0.00001x^2$	0.846	0.715	0.001	15.070	0.001
	Cubic	$y=0.0000+0.0167x-0.0000x^2+0.00001x^3$	0.717	0.514	0.003	6.335	0.013
MR	Quadratic	$y=59.2824+0.0012x-0.00001x^2$	0.974	0.949	0.000	111.374	0.000
	Cubic	$y=0.0000+0.0054x-0.0000x^2+0.00001x^3$	0.964	0.929	0.000	78.531	0.000
SA	Quadratic	$y=76.3757+0.0010x-0.00001x^2$	0.837	0.700	0.001	14.026	0.001
	Cubic	$y=0.0000+0.0048x-0.0000x^2+0.00001x^3$	0.701	0.491	0.001	5.792	0.017
*P*	Quadratic	$y=23.4735+0.0005x-0.00001x^2$	0.974	0.949	0.000	111.179	0.000
	Cubic	$y=0.0000+0.0021x-0.0000x^2+0.00001x^3$	0.964	0.929	0.000	78.681	0.000
MV	Quadratic	$y=187.5221+0.0027x-0.00001x^2$	0.962	0.925	0.001	74.304	0.000
	Cubic	$y=0.0000+0.0154x-0.0000x^2+0.00001x^3$	0.927	0.860	0.002	36.825	0.000

**Fig. 3:**
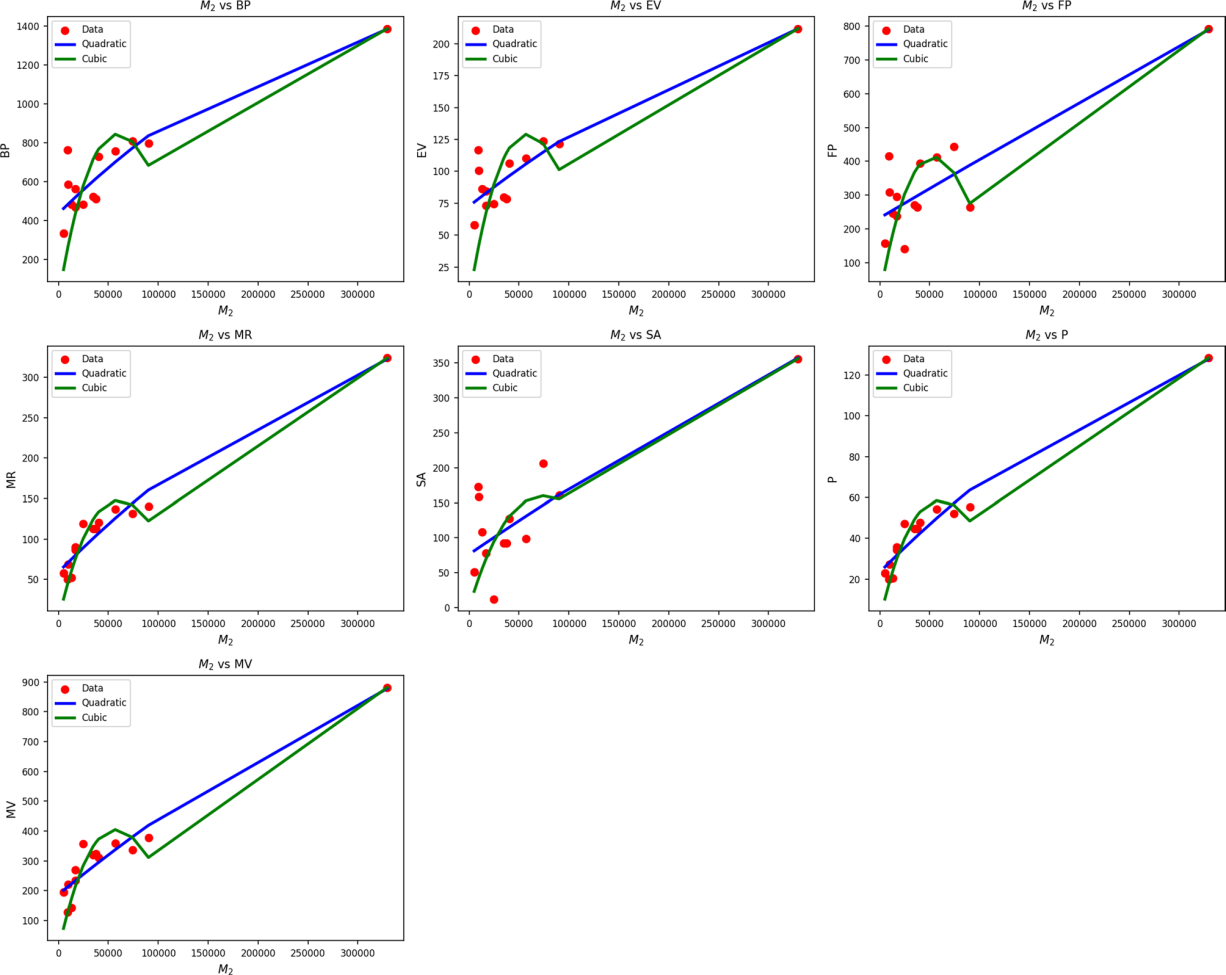
Scatter plots of actual data points (red) and regression model fits (Quadratic in blue, cubic in green) for various drug response parameters versus ${M_2(G)}$.

Table 5 also evaluates the association between $M_2(G)$ and drug response metrics through quadratic and cubic regression models. Quadratic models systematically perform better than their cubic counterparts, as indicated by superior R values (0.837 to 0.974) and significantly higher $R^2$ values. Cubic BP, EV, and FP models are weak in comparison, with$R^2$ as low as 0.184 being achieved by EV, implying weak explanatory capacity of this predictor in the cubic models. The standard error is also lower in almost all of the quadratic models, reflecting improved predictive accuracy. F-statistics further support the reliability of the quadratic models, with particularly high values observed in the cases of MR, P, and MV, indicating strong general significance. All p-values of the quadratic models are well below 0.001, affirming their statistical validity, while several cubic model p-values meet or exceed 0.05, diminishing their credibility. Figure 3 confirms the statistical results through visual regression fits on actual data points. The blue curves of the quadratics closely follow the red data points, especially in sequences like MR, P, and MV, where alignment is nearly flawless. In contrast, the green cubic curves tend to deviate unnecessarily, producing poor fits in data-sparse areas. The visual confirmation supports the numerical results, thereby establishing that drug response as a function of $M_2(G)$ is better represented by quadratics than cubics, through improved fit and reduced error rate.

**Table 6:** Statistical parameters and regression models for *FN*(*G*).

Property	$\text {Model}$	$\text {Equation}$	*R*	$R^2$	$S_E$	*F*	$p\text {-value}$
BP	Quadratic	$y=329.5860+0.1164x+0.00001x^2$	0.924	0.853	0.063	34.799	0.000
	Cubic	$y=205.7136+0.2676x-0.0000x^2+0.00001x^3$	0.926	0.858	0.246	22.185	0.000
EV	Quadratic	$y=62.0169+0.0114x+0.00001x^2$	0.922	0.849	0.009	33.798	0.000
	Cubic	$y=50.5625+0.0254x-0.0000x^2+0.00001x^3$	0.923	0.851	0.037	21.000	0.000
FP	Quadratic	$y=199.1664+0.0266x+0.00001x^2$	0.854	0.729	0.053	16.127	0.000
	Cubic	$y=15.6864+0.2505x-0.0001x^2+0.00001x^3$	0.871	0.758	0.200	11.507	0.001
MR	Quadratic	$y=41.9793+0.0214x+0.00001x^2$	0.967	0.934	0.011	85.184	0.000
	Cubic	$y=-21.6743+0.0990x-0.0000x^2+0.00001x^3$	0.977	0.955	0.036	77.880	0.000
SA	Quadratic	$y=66.3020+0.0099x+0.00001x^2$	0.856	0.732	0.027	16.412	0.000
	Cubic	$y=93.7077-0.0235x+0.0000x^2-0.00001x^3$	0.857	0.735	0.107	10.160	0.002
*P*	Quadratic	$y=16.6171+0.0085x+0.00001x^2$	0.966	0.934	0.004	85.064	0.000
	Cubic	$y=-8.6261+0.0393x-0.0000x^2+0.00001x^3$	0.977	0.955	0.014	77.744	0.000
MV	Quadratic	$y=166.9249+0.0298x+0.00001x^2$	0.951	0.904	0.035	56.802	0.000
	Cubic	$y=5.2872+0.2270x-0.0001x^2+0.00001x^3$	0.961	0.924	0.123	44.394	0.000

**Fig. 4:**
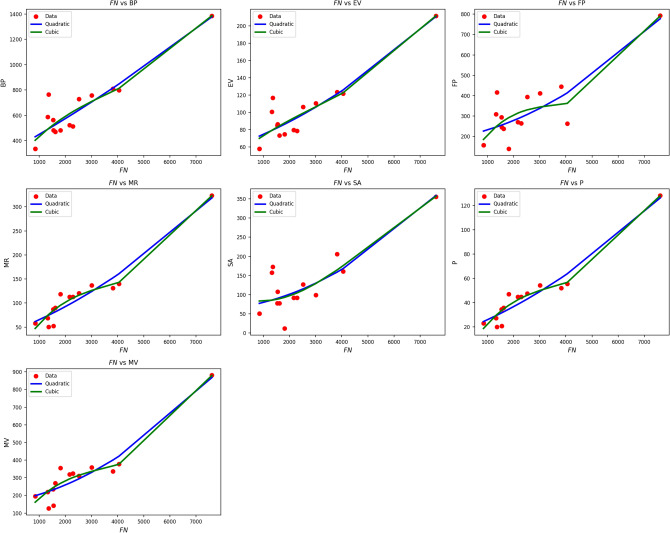
Scatter plots of actual data points (red) and regression model fits (quadratic in blue, cubic in green) for various drug response parameters versus *FN*(*G*).

Table 6 explores the impact of FN(G) on drug response criteria using both quadratic and cubic regression models. Quadratic models are highly predictive, with R values between 0.854 and 0.967, and $R^2$ values well over 0.72. Notably, the MR and P parameters exhibit $R^2$ values above 0.93, reflecting excellent explanatory capability. Although the cubic models produce slight improvements in R and $R^2$ for some variables such as MR (increasing from 0.934 to 0.955) and FP (from 0.729 to 0.758) these gains are offset by increased standard deviations, which signify reduced precision. All models yield statistically significant results, with p-values well below 0.005. F-measures are high, especially for MR and P, highlighting the robustness of the models. Overall, quadratic models offer a good trade-off between high correlation, low error, and simplicity, making them the preferred method when studying FN(G). Figure 4 provides a visual comparison of actual data points versus model projections for FN(G). The red data points are closely matched by the blue quadratic curve in most cases, particularly for MR, P, and MV, thereby replicating the superior statistical outcome. The cubic curves (green) offer minor improvements in curvature but tend to overfit in sparse data regions. Overall, the visual correspondence and consistent statistical results confirm that quadratic modeling is highly successful and superior to cubic modeling when analyzing drug response versus FN(G).

**Table 7:** Statistical parameters and regression models for $ND_3(G)$.

Property	$\text {Model}$	$\text {Equation}$	*R*	$R^2$	$S_E$	*F*	$p\text {-value}$
BP	Quadratic	$y=354.5080+0.0167x+0.0001x^2$	0.924	0.853	0.010	34.932	0.000
	Cubic	$y=161.5741+0.0548x-0.0000x^2+0.0001x^3$	0.933	0.870	0.033	24.611	0.000
EV	Quadratic	$y=64.7702+0.0016x+0.0001x^2$	0.924	0.854	0.001	35.211	0.000
	Cubic	$y=42.9374+0.0059x-0.0000x^2+0.0001x^3$	0.930	0.865	0.005	23.424	0.000
FP	Quadratic	$y=211.2776+0.0032x+0.0001x^2$	0.847	0.718	0.009	15.264	0.001
	Cubic	$y=1.9368+0.0446x-0.0000x^2+0.0001x^3$	0.877	0.769	0.028	12.214	0.001
MR	Quadratic	$y=54.0543+0.0023x+0.0001x^2$	0.952	0.907	0.002	58.443	0.000
	Cubic	$y=-18.6562+0.0166x-0.0000x^2+0.0001x^3$	0.971	0.943	0.006	60.847	0.000
SA	Quadratic	$y=69.4384+0.0013x+0.0001x^2$	0.865	0.748	0.004	17.790	0.000
	Cubic	$y=72.8962+0.0006x+0.0000x^2-0.0001x^3$	0.865	0.748	0.015	10.875	0.001
*P*	Quadratic	$y=21.4046+0.0009x+0.0001x^2$	0.952	0.907	0.001	58.381	0.000
	Cubic	$y=-7.4252+0.0066x-0.0000x^2+0.0001x^3$	0.971	0.943	0.002	60.755	0.000
MV	Quadratic	$y=198.7234+0.0015x+0.0001x^2$	0.937	0.879	0.006	43.494	0.000
	Cubic	$y=17.8818+0.0372x-0.0000x^2+0.0001x^3$	0.954	0.911	0.019	37.491	0.000

**Fig. 5:**
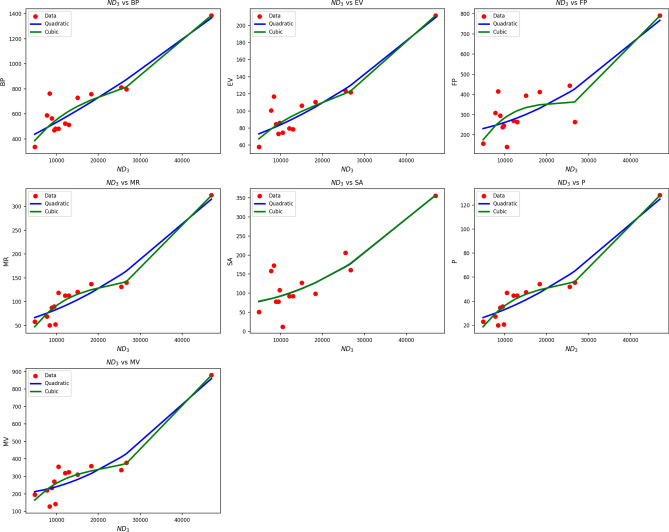
Scatter plots of actual data points (red) and regression model fits (Quadratic in blue, cubic in green) for various drug response parameters versus ${ND_3(G)}$.

Table 7 shows regression statistics relating drug response parameters to $ND_3(G)$ using both quadratic and cubic modeling. Both models show excellent fits, with high R values (quadratic: 0.847 to 0.952; cubic: up to 0.971) and significant $R^2$ values, indicating high levels of correlation. Notably, MR and P have $R^2$ values of 0.907 to 0.943, implying that $ND_3(G)$ can be used as a reliable predictor for these endpoints. While in some cases cubic models can improve $R^2$, e.g., MR (0.943 vs. 0.907) they tend to be associated with greater standard error. For example, SE increases from 0.002 to 0.006 for MR, which can reduce predictive consistency. p-values are highly significant across all variables $(p < 0.001)$, verifying the quality of both models. Nevertheless, considering the trade-off between model complexity and predictive performance, the quadratic models have the advantage of being more stable and interpretable, with fewer parameters and consistently strong performance across drug response types. Figure 5 schematically confirms the quantitative findings. The blue quadratic curves closely follow the red data points in all response parameters, especially for MR, P, and MV, with high fidelity to observation. The green cubic curves, though slightly more adaptable, diverge in less densely covered regions, where overfitting becomes more likely. This corroborates the conclusion that quadratic models provide accurate, transferable predictions by $ND_3(G)$ while remaining simple and precise in describing the drug response profile.

**Table 8:** Statistical parameters and regression models for $ND_5(G)$.

Property	$\text {Model}$	$\text {Equation}$	*R*	$R^2$	$S_E$	*F*	$p\text {-value}$
BP	Quadratic	$y=325.7070+3.8796x+0.0054x^2$	0.899	0.809	2.785	25.425	0.000
	Cubic	$y=483.3927-2.8274x+0.0823x^2-0.0002x^3$	0.901	0.812	15.530	15.875	0.000
EV	Quadratic	$y=63.9238+0.3223x+0.0018x^2$	0.897	0.804	0.411	24.584	0.000
	Cubic	$y=95.7022-1.0293x+0.0173x^2-0.0001x^3$	0.900	0.810	2.272	15.646	0.000
FP	Quadratic	$y=209.5205+0.5680x+0.0102x^2$	0.845	0.714	2.125	14.966	0.001
	Cubic	$y=137.0239+3.6516x-0.0252x^2+0.0001x^3$	0.846	0.716	11.917	9.227	0.002
MR	Quadratic	$y=17.8676+1.2233x+0.0010x^2$	0.988	0.976	0.252	247.548	0.000
	Cubic	$y=-39.0077+3.6424x-0.0268x^2+0.0001x^3$	0.991	0.983	1.210	209.590	0.000
SA	Quadratic	$y=82.2405-0.0540x+0.0064x^2$	0.824	0.680	1.146	12.728	0.001
	Cubic	$y=224.4767-6.1039x+0.0758x^2-0.0002x^3$	0.840	0.706	6.175	8.812	0.003
*P*	Quadratic	$y=7.0570+0.4849x+0.0004x^2$	0.988	0.976	0.100	246.748	0.000
	Cubic	$y=-15.5173+1.4450x-0.0106x^2+0.0001x^3$	0.991	0.983	0.481	208.846	0.000
MV	Quadratic	$y=100.1928+2.4029x+0.0059x^2$	0.975	0.951	0.962	115.803	0.000
	Cubic	$y=-67.2327+9.5241x-0.0758x^2+0.0002x^3$	0.979	0.959	4.951	85.269	0.000

**Fig. 6:**
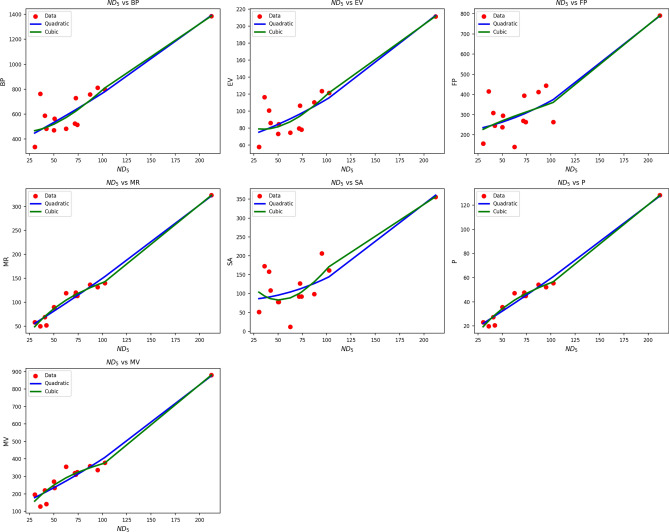
Scatter plots of actual data points (red) and regression model fits (quadratic in blue, cubic in green) for various drug response parameters versus *NH*(*G*).

Table 8 assesses the association between $ND_5(G)$ and drug response parameters through quadratic and cubic regression analysis. Both models generate high R and $R^2$ values, indicating strong associations across all parameters. Of particular note are the very high $R^2$ values of MR and P in both models 0.976 in the quadratic and 0.983 in the cubic implying very high predictive capability. Nevertheless, cubic models tend to have greater standard errors, which may imply overfitting. The SE, for example, increases significantly from 2.785 (quadratic) to 15.530 (cubic) in the case of BP, and the same pattern is observed in other parameters such as EV and FP. Although the cubic models increase $R^2$ slightly, the accompanying rise in SE tends to reduce their usefulness in practice. All models retain high F-values and statistically highly significant p-values $(p < 0.005)$, endorsing their validity. However, since the improvement in explanatory ability is modest and the increase in error is greater, the quadratic models present a superior trade-off between precision and simplicity in most drug response prediction contexts involving $ND_5(G)$. Figure 6 provides supporting evidence with clear visualizations. The quadratic model (blue line) closely follows the pattern of actual data points (red), especially for MR, P, and MV, where the fit is nearly exact. The cubic model (green) may offer more flexibility but oscillates excessively in low-density regions, indicating instability. Overall, both visual and statistical evidence consistently indicate the quadratic model as the more useful and stable choice for modeling drug responses.

**Table 9:** Statistical parameters and regression models for *NH*(*G*).

Property	$\text {Model}$	$\text {Equation}$	*R*	$R^2$	$S_E$	*F*	$p\text {-value}$
BP	Quadratic	$y=386.2460+25.9214x+2.0810x^2$	0.871	0.759	41.999	18.886	0.000
	Cubic	$y=1120.3201-344.0972x+53.6588x^2-1.9232x^3$	0.889	0.790	291.420	13.816	0.000
EV	Quadratic	$y=74.9076+0.3131x+0.4790x^2$	0.873	0.761	6.077	19.133	0.000
	Cubic	$y=191.8350-58.6256x+8.6946x^2-0.3063x^3$	0.894	0.799	41.496	14.566	0.000
FP	Quadratic	$y=261.7664-9.6184x+2.4890x^2$	0.828	0.685	29.933	13.034	0.001
	Cubic	$y=398.8229-78.7033x+12.1189x^2-0.3591x^3$	0.829	0.688	221.690	8.070	0.004
MR	Quadratic	$y=6.6778+16.3706x+0.1551x^2$	0.995	0.990	2.172	610.537	0.000
	Cubic	$y=-53.9700+46.9408x-4.1061x^2+0.1589x^3$	0.997	0.993	13.206	560.339	0.000
SA	Quadratic	$y=119.1097-11.5839x+1.5634x^2$	0.803	0.645	16.198	10.892	0.002
	Cubic	$y=418.6505-162.5709x+22.6099x^2-0.7848x^3$	0.835	0.697	111.390	8.415	0.003
*P*	Quadratic	$y=2.6200+6.4889x+0.0616x^2$	0.995	0.990	0.864	606.386	0.000
	Cubic	$y=-21.4871+18.6404x-1.6323x^2+0.0632x^3$	0.997	0.993	5.255	556.116	0.000
MV	Quadratic	$y=63.3543+35.3380x+0.8079x^2$	0.988	0.976	9.006	244.300	0.000
	Cubic	$y=-170.9294+153.4315x-15.6535x^2+0.6138x^3$	0.991	0.983	56.533	211.194	0.000

**Fig. 7:**
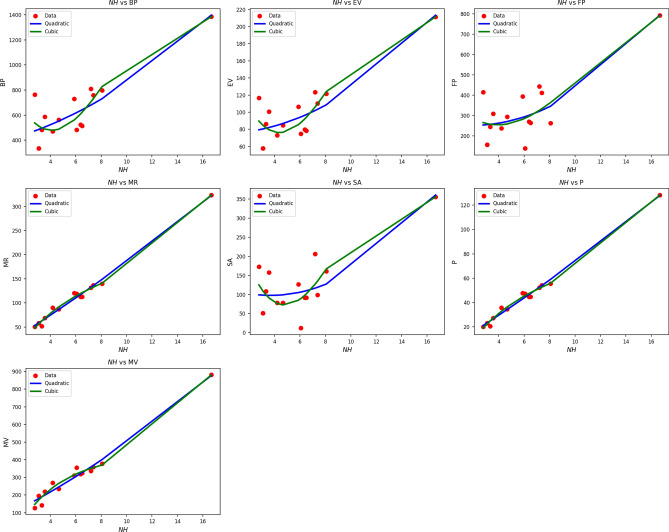
Scatter plots of actual data points (red) and regression model fits (quadratic in blue, cubic in green) for various drug response parameters versus *NH*(*G*).

Table 9 shows regression results of NH(G) on drug response measures with quadratic and cubic models. Both model forms produce statistically significant results, with p-values less than 0.005 and high F-statistics across the board. Parameters like MR, P, and MV have very high R values of up to 0.995 and 0.997, indicating high correlation. Cubic models improve $R^2$ values slightly, such as when MR increases to 0.993 from 0.990, but this tends to come at the expense of greater standard error. SE, for instance, increases dramatically from 41.999 in the quadratic form to 291.420 in the cubic form, meaning less reliable estimations. Overall, while cubic forms can better fit nonlinear curves, they add more variability and complexity without providing proportional increases in fit. Therefore, quadratic models generate more stable, interpretable, and more efficient solutions for assessing the impact of drug responses by NH(G). Figure 7 reinforces these results. Quadratic fits (blue lines) closely follow actual data (red squares) for most parameters, e.g., MR, MV, and P. Cubic models (green lines) diverge or over-curve in some areas, particularly where data are sparse or less variable. These over-swings indicate overfitting in some data sets, even with slight $R^2$ improvements. Both visually and statistically, the quadratic model offers a smoother, more regular portrayal of the drug response behavior associated with NH(G) and, as such, represents the better option when performing predictive modeling in this genomic context.

**Table 10:** Statistical parameters and regression models for *NI*(*G*).

Property	$\text {Model}$	$\text {Equation}$	*R*	$R^2$	$S_E$	*F*	$p\text {-value}$
BP	Quadratic	$y=355.8127+2.4398x+0.0045x^2$	0.908	0.825	1.861	28.214	0.000
	Cubic	$y=380.2739+1.6293x+0.0116x^2-0.0001x^3$	0.908	0.825	8.461	17.260	0.000
EV	Quadratic	$y=67.2233+0.1854x+0.0012x^2$	0.905	0.819	0.275	27.240	0.000
	Cubic	$y=77.8100-0.1654x+0.0043x^2-0.0001x^3$	0.906	0.821	1.244	16.802	0.000
FP	Quadratic	$y=218.1404+0.2769x+0.0063x^2$	0.845	0.714	1.482	14.964	0.001
	Cubic	$y=97.7782+4.2650x-0.0284x^2+0.0001x^3$	0.851	0.723	6.626	9.590	0.002
MR	Quadratic	$y=32.9614+0.6772x+0.0013x^2$	0.981	0.963	0.221	154.388	0.000
	Cubic	$y=-23.7449+2.5561x-0.0151x^2+0.0001x^3$	0.987	0.975	0.821	143.624	0.000
SA	Quadratic	$y=84.5140-0.0991x+0.0039x^2$	0.835	0.697	0.777	13.824	0.001
	Cubic	$y=162.3024-2.6765x+0.0263x^2-0.0001x^3$	0.844	0.713	3.442	9.100	0.003
*P*	Quadratic	$y=13.0414+0.2684x+0.0005x^2$	0.981	0.963	0.088	154.014	0.000
	Cubic	$y=-9.4545+1.0137x-0.0060x^2+0.0001x^3$	0.987	0.975	0.326	143.202	0.000
MV	Quadratic	$y=139.6352+1.1535x+0.0053x^2$	0.966	0.934	0.777	84.795	0.000
	Cubic	$y=-14.8241+6.2714x-0.0393x^2+0.0001x^3$	0.973	0.947	3.158	65.801	0.000

**Fig. 8:**
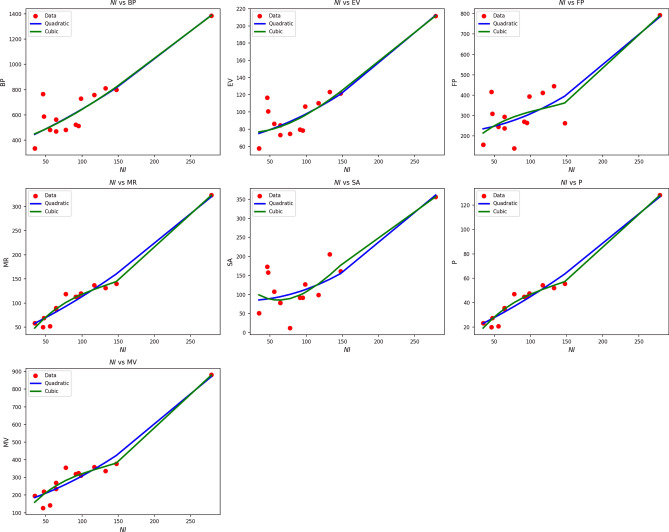
Scatter plots of actual data points (red) and regression model fits (quadratic in blue, cubic in green) for various drug response parameters versus *NI*(*G*).

Table 10 assesses the impact of NI(G) on different drug response parameters through both the quadratic and cubic regression models. The findings reveal excellent fits of the models to all responses, with R values well over 0.90 and $R^2$ well over 0.82 in most of the quadratic models. Notably, MR, P, and MV show very good predictability with quadratic $R^2$ values of 0.963, 0.963, and 0.934, respectively. Although the cubic models improve $R^2$ slightly, e.g., to 0.975 in the case of MR, the concomitant increase in standard error lowers their predictive clarity. The SE, for example, rises from 1.861 to 8.461 when moving from quadratic to cubic, respectively. Although both models yield highly significant p-values $(p < 0.005)$, the SE levels are consistently lower in the quadratic models, implying greater predictive reliability with reduced risk of overfitting. F-values are particularly large for MR, P, and MV, confirming the robustness of both models. Nevertheless, the superior trade-off between simplicity, accuracy, and stability makes the quadratic form the better choice to represent the impact of NI(G). Figure 8 reinforces these findings visually. Quadratic curves (blue) closely follow the red actual data points, particularly in the cases of MR, P, and MV, where the fits are tight and stable. Cubic fits (green), while more flexible, tend to veer off or oscillate more widely, especially in regions of sparse data. Both visual and statistical analyses, therefore, verify the quadratic model as the most reliable paradigm for representing drug responses as a function of NI(G), offering strong, stable, and meaningful results.

## Random forest regression analysis

Random Forest (RF) regression was utilized in the present work to predict molecular properties by employing several topological indices as input variables. Random Forest is an ensemble learning method that constructs multiple decision trees during training and outputs the average prediction, thereby enhancing accuracy and minimizing overfitting. Topological indices representing the structural attributes of molecules were used as predictors, while molecular attributes such as boiling point, molar refractivity, and polarizability were treated as dependent variables. The performance of the Random Forest model was evaluated using several statistical measures. The proportion of variance in the dependent variable explained by the independent variables is referred to as the coefficient of determination ($R^2$) and is defined as:

$$R^2 = 1 - \frac{\sum _{i=1}^{n}(y_i - \hat{y}_i)^2}{\sum _{i=1}^{n}(y_i - \bar{y})^2},$$

The Mean Absolute Error (MAE) is defined as:

$${MAE} = \frac{1}{n} \sum _{i=1}^{n} \left| y_i - \hat{y}_i\right| ,$$

The Root Mean Square Error (RMSE) measures the standard deviation of prediction errors and is calculated by:

$${RMSE} = \sqrt{\frac{1}{n} \sum _{i=1}^{n} (y_i - \hat{y}_i)^2}$$,

where $y_i$ is the true value, $\hat{y}_i$ is the estimated value, $\bar{y}$ of the true values, and $n$ is the number of observations. These metrics collectively provide an overall assessment of the model s accuracy, the magnitude of errors, and its ability to explain the relationship between topological indices and molecular properties.

**Fig. 9:**
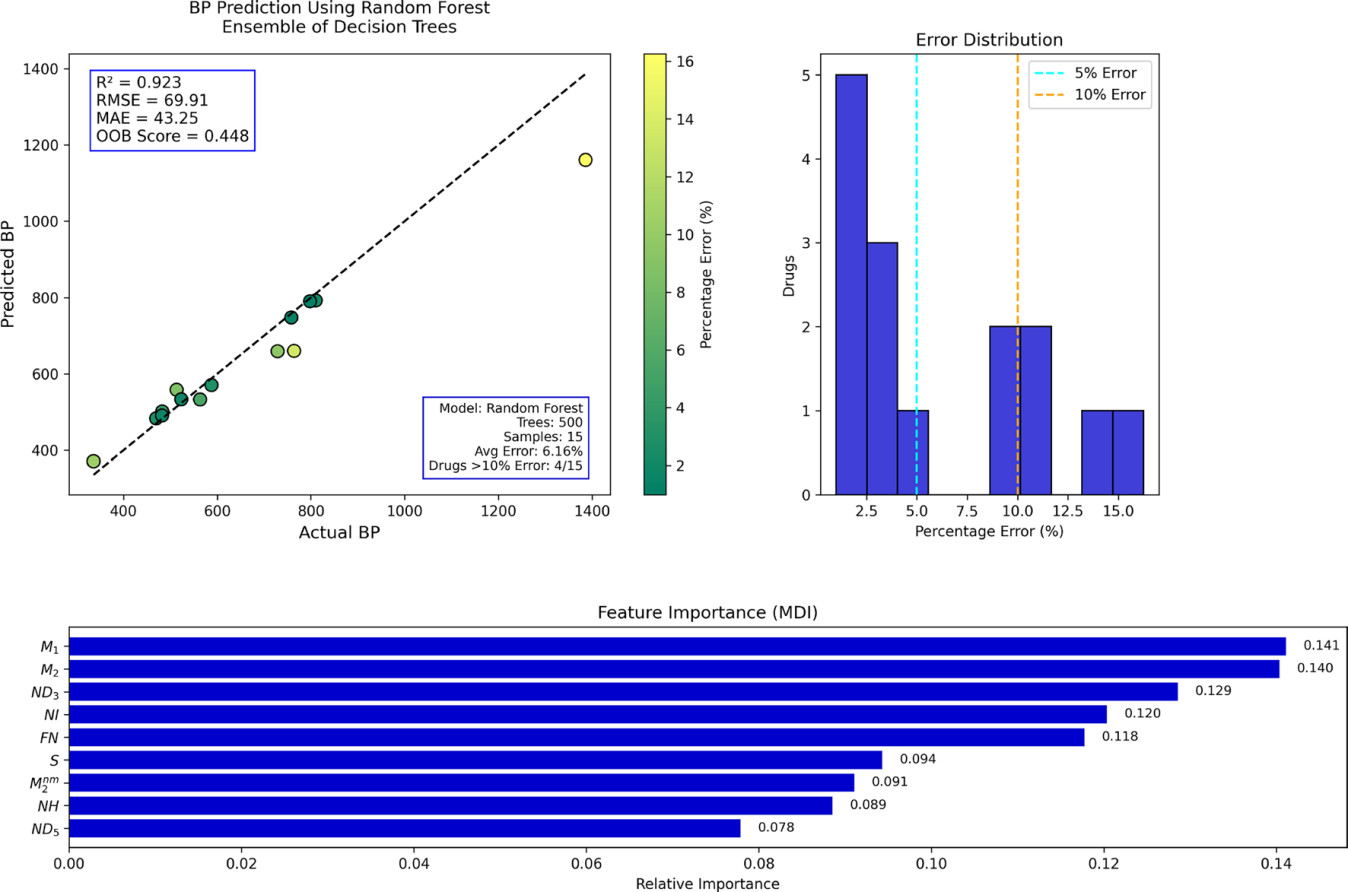
RF regression model comparing actual vs. predicted BP based on the indices.

**Fig. 10:**
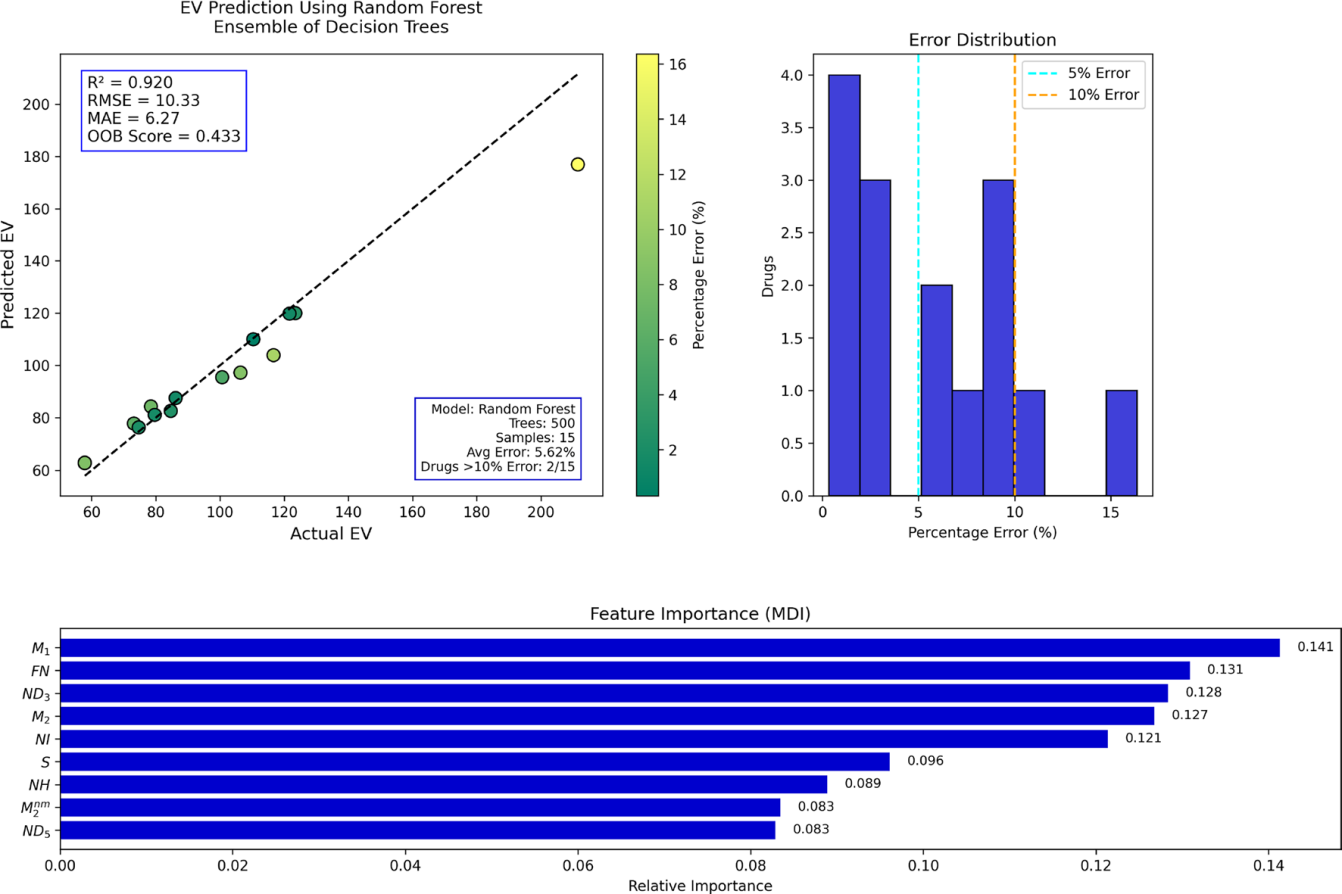
RF regression model comparing actual vs. predicted EV based on the indices.

**Fig. 11:**
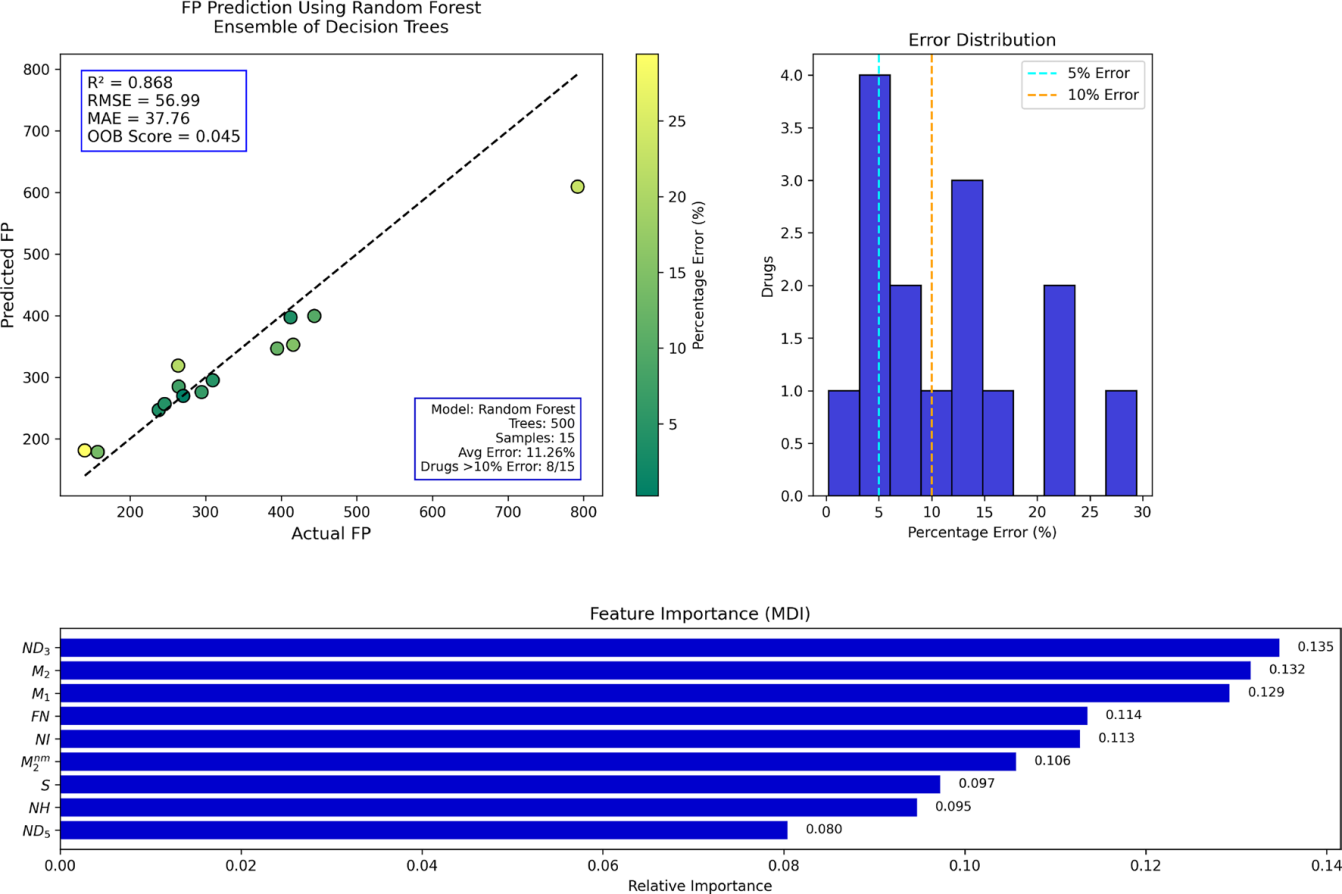
RF regression model comparing actual vs. predicted FP based on the indices.

**Fig. 12:**
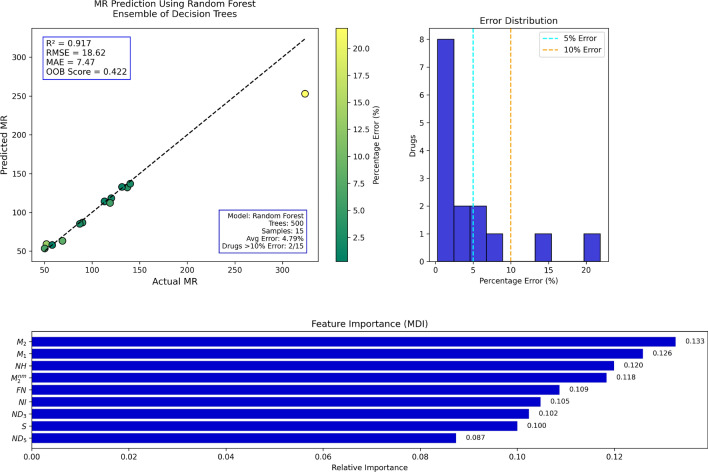
RF regression model comparing actual vs. predicted MR based on the indices.

**Fig. 13:**
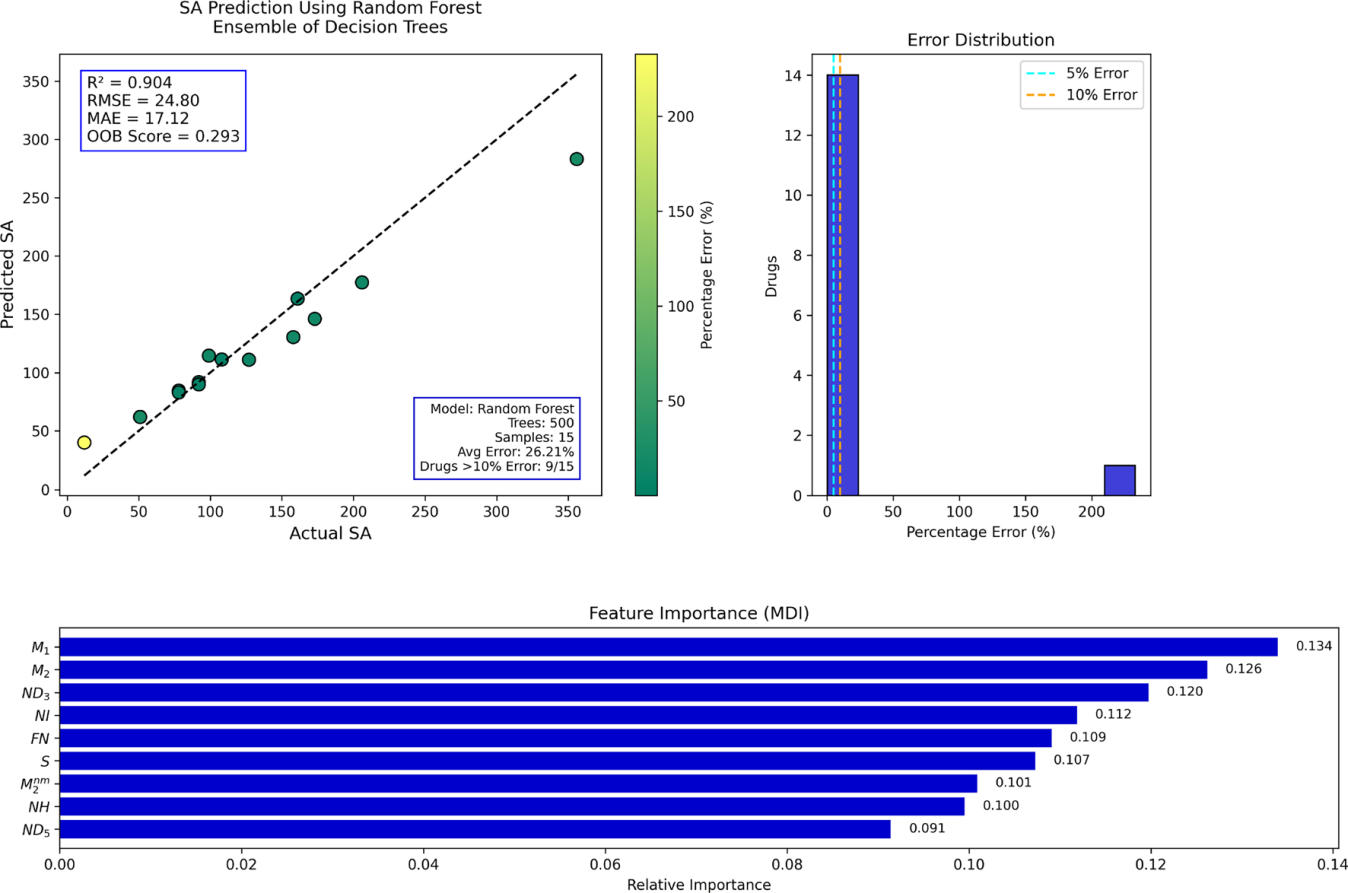
RF regression model comparing actual vs. predicted SA based on the indices.

**Fig. 14:**
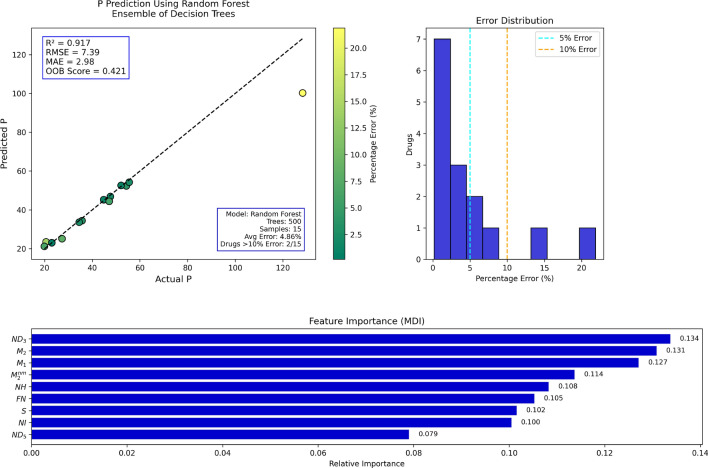
RF regression model comparing actual vs. predicted P based on the indices.

**Fig. 15:**
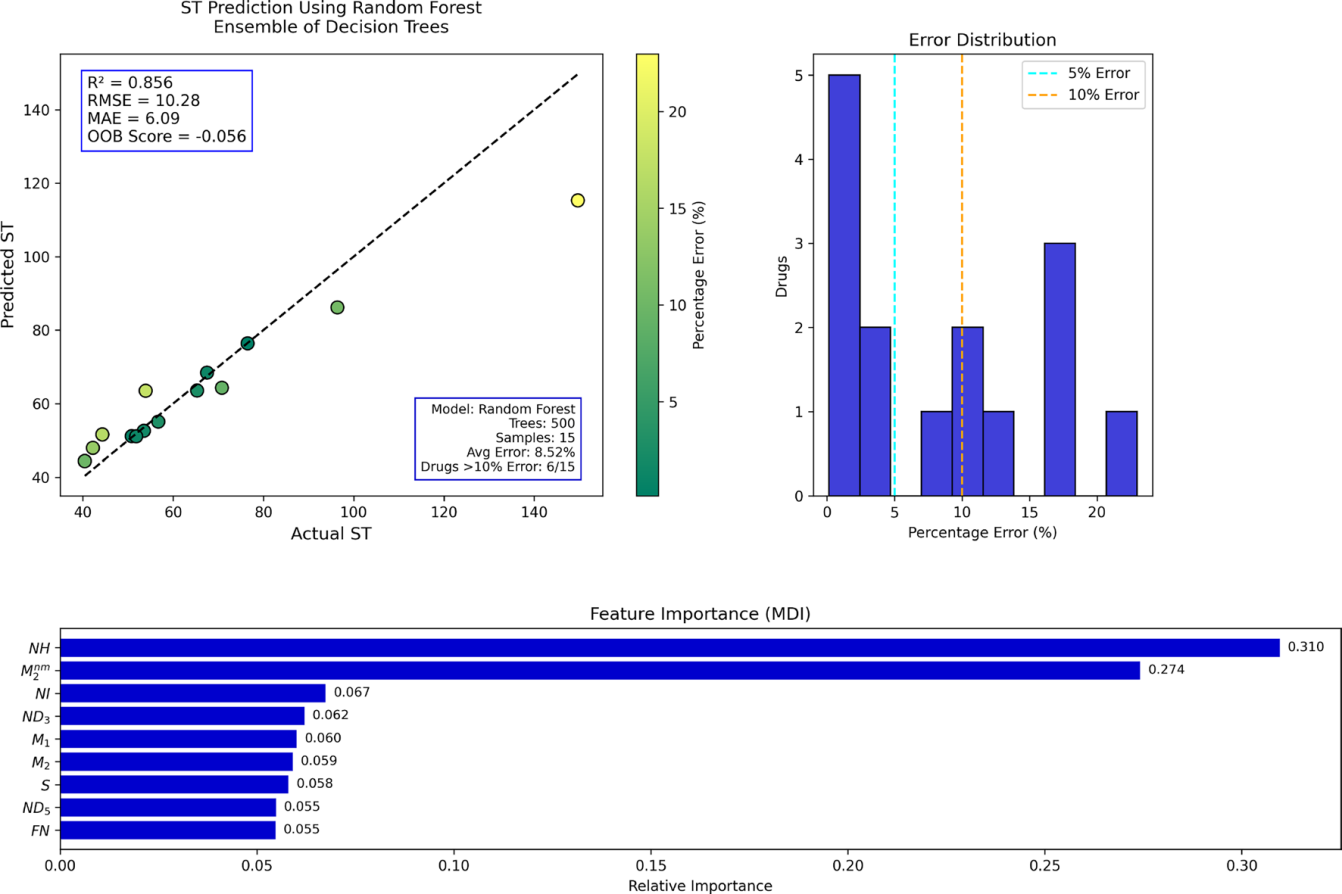
RF regression model comparing actual vs. predicted ST based on the indices.

**Fig. 16:**
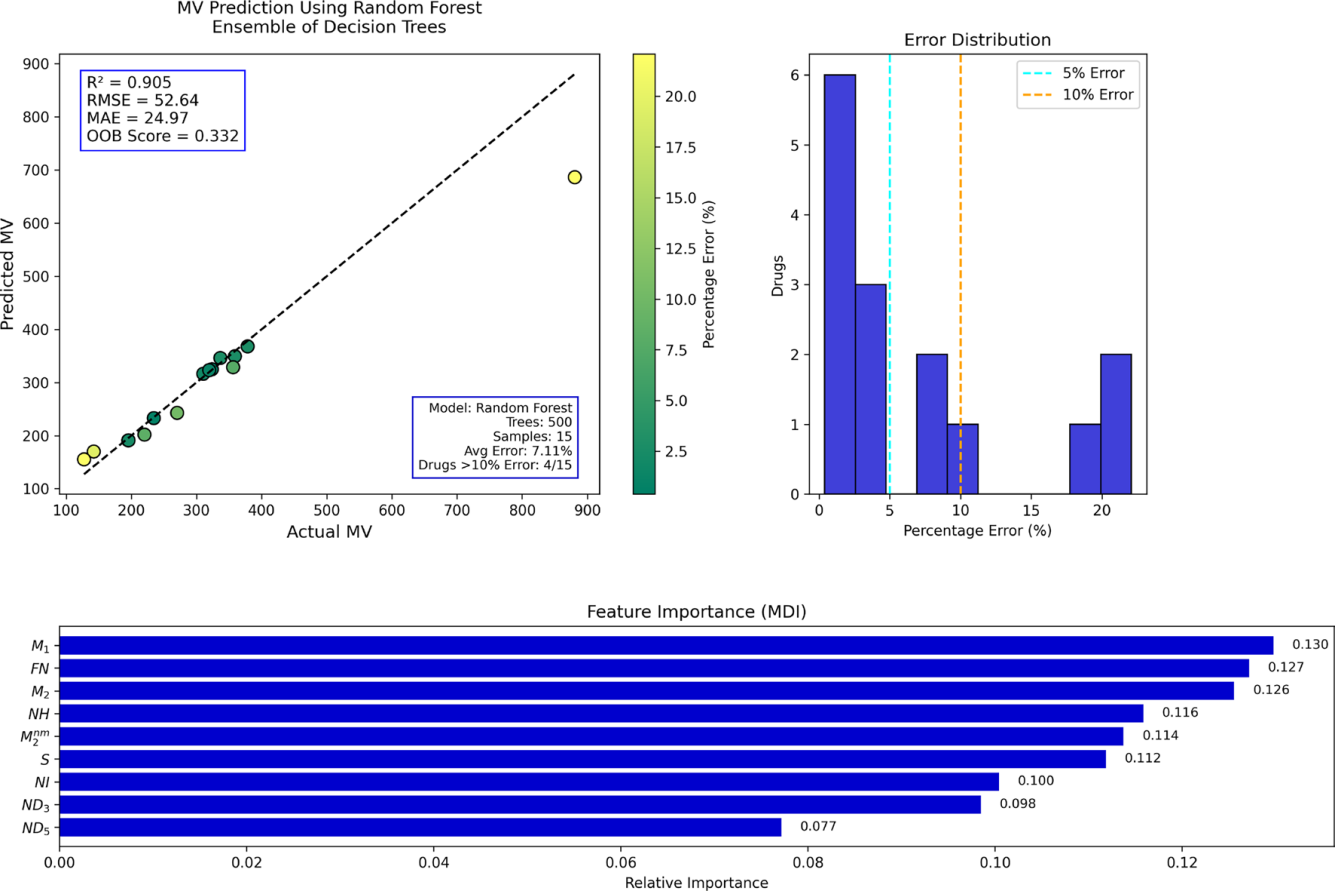
RF regression model comparing actual vs. predicted MV based on the indices.

- *Boiling point prediction using random forest:* Table 11 depicts the actual versus Random Forest-model-predicted boiling points. Overall, the percentages of errors are low, and the performance of the model is presumably good. The majority of the deviations are less than 10%, with the exception of some high-error outliers such as the first and final entries. This suggests that although the model is generally trustworthy, it can perform poorly on compounds with unusual boiling points or highly complex structures.
  Figure 9 plots the actual versus predicted boiling temperatures. The majority of the data points are tightly clustered around the ideal prediction line, which verifies a very strong correlation. Minor deviations toward outliers reveal some loss of accuracy at higher or lower boiling ranges; yet, the overall prediction quality remains high.
- *Enthalpy of vaporization prediction using random forest:* Table 12 summarizes the actual and predicted enthalpy of vaporization values. The percentage error is always less than 10%, except in the first and last entries. This indicates that the Random Forest model is generally effective for this property, with the exception of predicting some edge cases, which could be improved.
  Figure 10 shows that the majority of predicted values are fairly close to the actual values, aligning along the ideal correlation line. Small deviations indicate the model s shortcomings in capturing subtle differences, but the overall predictive pattern confirms that the model is appropriate for predicting enthalpy of vaporization.
- *Flash point prediction using random forest:* Table 13 shows greater variability in the accuracy of flash point value predictions. There are several entries, especially the first and the last, with errors greater than 20%. These indicate that flash point could be more structure-sensitive or less well described by the present topological descriptors.
  Figure 11 demonstrates a broader spread around the diagonal prediction line, especially towards higher flash point values. This reflects decreased reliability of prediction compared to the previous characteristics. The RF predictor identifies prevailing trends but lags in accuracy for some compounds.
- *Molar refractivity prediction using random forest:* Highly precise molar refractivity predictions are demonstrated in Table 14. The majority of the errors are less than 5%, with the first one being the only exception. The findings indicate that the topological indices employed are well related to molar refractivity, allowing the model to predict confidently throughout the dataset.
  Figure 12 confirms the model s precision, with points tightly grouped around the ideal prediction line. Few deviations are observed, which supports the model s high performance for molar refractivity estimation using structural descriptors.
- *Surface area prediction using random forest:* In Table 15, the prediction errors in surface area are more variable than in the previous examples. There are several entries over 10%, and one instance has a huge error over 200%, reflecting considerable deviation for certain compounds. The structural influences on surface area may not be adequately represented within the model.
  Figure 13 shows varying prediction accuracy, with individual results straying significantly from the ideal line. Mid-range results are acceptable, but outer values indicate instability of the model or lack of relevance for the descriptor in determining surface area.
- *Polarizability prediction using random forest:* Table 16 provides generally correct polarizability predictions, with errors less than 5% in most entries. There are some outliers, particularly at the extremes of the lowest and highest values. The result is that the RF model performs well on moderate values but should be adjusted for boundary conditions.
  Figure 14 depicts high correlation between calculated and experimental polarizability values. The majority of the data lie along the ideal line, supporting the validity of the model. Minor outliers at high values indicate slight deviation in prediction, yet global performance is strong.
- *Surface tension prediction using random forest:* Table 17 depicts mixed performance in the prediction of surface tension. The majority of the values have 10 20% error, with maximum error being almost 23%. The outcomes indicate moderate accuracy with some constraints in the prediction of higher values of surface tension accurately.
  Figure 15 demonstrates reasonable clustering along the ideal line, but with greater spreading than in previous figures. This indicates a decrease in predictive accuracy for surface tension. The model follows trends well, yet some variability exists.
- *Molar volume prediction using random forest:* Table 18 shows variable prediction accuracy in molar volume, with errors ranging from less than 1% to more than 22%, notably in the highest and lowest actual values. The model performs well in generalizing mid-range values but loses fidelity toward the extremes.
  Figure 16 displays good correspondence between predicted and observed values. Although most data cluster along the ideal line, outliers show large discrepancies. The RF model describes broad tendencies but is less accurate when molar volumes are very large or very small.

**Table 11:** Actual and predicted values of BP.

Actual	1386	469.7	758.1	336.1	810.3	798.1	482.7	587.8	336.1	563.5	728.8	513.4	523.3	482.3	764
Predicted	1160.71	483.21	747.57	371.28	792.44	790.26	501.35	570.52	371.28	532.77	659.15	558.26	533.27	490.67	660.14
Error (%)	16.25	2.88	1.39	10.47	2.20	0.98	3.86	2.94	10.47	5.45	9.56	8.74	1.90	1.74	13.59

**Table 12:** Actual and Predicted Values of EV.

Actual	211.5	73.2	110.4	57.9	123.5	121.7	86.2	100.7	57.9	84.7	106.4	78.5	79.7	74.7	116.7
Predicted	176.89	77.79	110.03	62.80	120.01	119.81	87.52	95.50	62.80	82.61	97.24	84.31	81.09	76.30	103.93
Error (%)	16.37	6.27	0.34	8.46	2.83	1.55	1.53	5.17	8.46	2.46	8.61	7.40	1.74	2.15	10.94

**Table 13:** Actual and Predicted Values of FP.

Actual	792.1	237.9	412.3	157.1	443.8	263.6	245.7	309.3	157.1	294.6	394.6	264.3	270.3	140.0	415.8
Predicted	609.36	246.73	397.22	178.63	399.41	318.82	256.48	295.09	178.63	275.89	346.58	284.77	269.68	181.19	352.76
Error (%)	23.07	3.71	3.66	13.70	10.00	20.95	4.39	4.59	13.70	6.35	12.17	7.75	0.23	29.42	15.16

**Table 14:** Actual and Predicted Values of MR.

Actual	323.7	90.0	137.0	58.1	131.5	140.1	52.1	68.9	58.1	87.1	120.2	113.1	113.1	118.9	50.3
Predicted	252.93	86.86	132.16	57.95	132.80	136.91	59.12	63.31	57.95	85.20	118.31	114.10	114.21	112.12	53.47
Error (%)	21.86	3.49	3.53	0.27	0.99	2.28	13.47	8.12	0.27	2.18	1.57	0.89	0.98	5.70	6.30

**Table 15:** Actual and Predicted Values of SA.

Actual	356	78	99	51	206	161	108	158	51	78	127	92	92	12	173
Predicted	283.13	84.53	114.48	61.92	177.32	163.37	111.20	130.39	61.92	82.95	110.98	91.76	89.81	39.95	146.07
Error (%)	20.47	8.37	15.64	21.42	13.92	1.47	2.96	17.47	21.42	6.35	12.62	0.27	2.38	232.88	15.56

**Table 16:** Actual and Predicted Values of P.

Actual	128.3	35.7	54.3	23.0	52.1	55.5	20.6	27.3	23.0	34.5	47.6	44.8	44.8	47.1	19.9
Predicted	100.23	34.41	52.32	23.04	52.59	54.24	23.49	25.12	23.04	33.64	46.86	45.16	45.25	44.38	21.19
Error (%)	21.88	3.62	3.66	0.17	0.94	2.27	14.03	7.99	0.17	2.50	1.55	0.81	0.99	5.78	6.47

**Table 17:** Actual and Predicted Values of ST.

Actual	53.9	42.2	67.5	44.3	96.4	76.5	65.3	70.8	44.3	53.5	56.7	50.8	51.8	40.4	149.7
Predicted	63.49	47.95	68.41	51.59	86.18	76.39	63.55	64.30	51.59	52.58	55.08	51.13	51.08	44.40	115.31
Error (%)	17.79	13.62	1.35	16.47	10.61	0.14	2.68	9.18	16.47	1.72	2.86	0.64	1.39	9.89	22.97

**Table 18:** Actual and Predicted Values of MV.

Actual	880.7	270.3	359.0	195.3	336.6	378.5	142.3	220.2	195.7	234.5	310.4	323.7	319.5	356.2	127.4
Predicted	686.31	242.76	349.33	190.93	346.04	367.87	169.99	201.84	191.10	232.91	316.37	324.96	323.20	328.67	155.24
Error (%)	22.07	10.19	2.69	2.24	2.80	2.81	19.46	8.34	2.35	0.68	1.92	0.39	1.16	7.73	21.85

## Conclusion

This work exhibits a meticulous analysis of the topological indices of degree-based neighborhoods that can be employed for forecasting the physicochemical features of anticancer agents through regression and machine learning techniques. After the construction of molecular graph structures for fifteen bone cancer agents and the calculations of their respective NM-polynomials, we measured a host of structural descriptors such as $M_1$, $M_2$, $FN$, $ND_3$, $ND_5$, $NH$, and $NI$. These indices were employed for quantitative structure property relationship (QSPR) modeling for the prediction of critical drug features such as boiling point, molar refractivity, surface area, and molecular volume. Among the explored predictive models, quadratic regression was better than cubic regression according to fit quality, lower standard error, and statistical significance. In addition, random forest models delivered stable results, which supported the cheminformatic promise of machine learning. It became clear that not only are topological indices based on neighborhoods mathematically meaningful but they are of high explanatory power for molecular property prediction. Visual inspection of regression fits, together with resulting statistics, supported the predictivity of the models.

*Future work*. can be pursued along some directions. In the first instance, expanding the dataset such that more diverse drug molecules are included could increase the level of generalizability. Second, studying other topological descriptors, for instance, distance- or spectral-based indices, could be more illuminating. Inclusion of 3D molecular data or hybrid descriptors could increase the level of predictivity. Also, employing deep learning models or ensemble methods could give better performance on larger sets. Finally, cross-validation of the outputs with experimental or clinical data would further confirm the approach’s real-world value, substantiating its use for early-stage drug discovery and design.

## Data Availability

The datasets used and/or analysed during the current study available from the corresponding author on reasonable request.
